# “I Have No Idea What's in It!”—A Qualitative Study of Adolescents' Conceptions of Milk Alternatives

**DOI:** 10.1002/fsn3.70259

**Published:** 2025-05-20

**Authors:** Lena Szczepanski, Insa Stonner, Florian Fiebelkorn

**Affiliations:** ^1^ Biology Didactics, Department of Biology/Chemistry Osnabrück University Osnabrück Germany

**Keywords:** consumer perception, dairy alternatives, nutrition education, sustainable nutrition, young people

## Abstract

This study aimed to analyze and compare adolescents' conceptions of plant‐based milk alternatives (PBMAs) and precision fermentation‐based milk alternatives (AFM), their ingredients, and production. For this purpose, a qualitative approach with semi‐structured interviews was chosen. A total of twenty‐five adolescents from Germany aged between 16 and 19 years were interviewed in September and October 2022. The interviews were analyzed using qualitative content analysis. The results show that the adolescents in our study held simplified conceptions of the ingredients (water, oats, sugar) and production (grind oats, mix with water, and heat the liquid if necessary) of PBMAs. Their conceptions differed depending on cow's milk and PBMA consumption, with PBMA consumers having the most differentiated conceptions. Regarding AFM, the adolescents held vague conceptions and expressed their lack of familiarity with the product. They either had no concept of AFM or believed AFM to be artificial, synthetic, or laboratory milk. In conclusion, adolescents' conceptions of milk alternatives were simplified and dependent on consumption patterns, with PBMA consumers having more differentiated conceptions. Based on our findings, we suggest that nutritional education should address the ingredients and production of cow's milk and milk alternatives, as well as their advantages and disadvantages, to enable young people to make informed decisions about their consumption of milk (alternatives).

## Graphical abstract

Adolescents from Germany held simplified conceptions of the ingredients (water, oats, sugar) and production (grind oats, mix with water, and heat the liquid if necessary) of plant‐based milk alternatives. Regarding animal‐free milk, adolescents from Germany held vague conceptions and expressed their lack of familiarity with the product. They either had no concept of AFM or believed AFM to be artificial, synthetic, or laboratory.

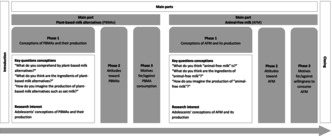

## 1 Introduction

Over the past decade, the consumption of plant‐based milk alternatives (PBMAs) has become increasingly popular in Europe, with oat, almond, and soy milk being the most consumed (European Union's Horizon 2020 Research and Innovation Programme [No 862957], 2021a, 2021b). The environmental impact of PBMAs is lower than that of cow's milk. For example, the production of 1 L of conventional cow's milk causes between 1.4 and 3.2 kg CO_2_ equivalents, while the production of 1 L of oat, almond, or soy milk causes between 0.4 and 1.0 kg CO_2_ equivalents (Carlsson Kanyama et al. 2021; Geburt et al. 2022; Poore and Nemecek 2019). Furthermore, the production of conventional cow's milk requires more land than the production of oat, almond, or soy milk (Carlsson Kanyama et al. 2021; Geburt et al. 2022; Poore and Nemecek 2019): Producing 1 L of conventional cow's milk requires 1 m^2^ of land, while producing 1 L of oat, almond, or soy milk requires between 0.4 and 0.6 m^2^ (Geburt et al. 2022). Water use is also lower in the production of oat and soy milk (1.7–2.7 m^3^/L) than in the production of conventional cow's milk (7.4 m^3^/L). Water use in the production of almond milk depends heavily on the region, though, and studies have shown both higher and lower values compared to cow's milk (Carlsson Kanyama et al. 2021; Geburt et al. 2022). Notably, the environmental impacts of cow's milk and PBMA production generally depend on the area of cultivation and the production method (organic or conventional; Carlsson Kanyama et al. 2021; Geburt et al. 2022; Poore and Nemecek 2019).

However, the nutritional values of PBMAs and cow's milk differ. The macro‐ and micronutrients in oat, almond, or soy milk are not equivalent to those in cow's milk, nor is the energy, fat, vitamin (e.g., A, B2, B12, and D), and other mineral (e.g., calcium, iodine, zinc, magnesium) content (Paul et al. 2019; Singh‐Povel et al. 2022). For example, 100 g of oat and almond milk have a lower protein content than 100 g of cow's milk, while the protein content of soy milk is roughly comparable to that of cow's milk (Paul et al. 2019; Pointke et al. 2022b; Singh‐Povel et al. 2022). Because cow's milk provides essential nutrients such as calcium and iodine, some manufacturers add minerals or vitamins to PBMAs (Reyes‐Jurado et al. 2021).

Companies worldwide are working on the production of another milk alternative—termed “animal‐free milk” (AFM)—that is molecularly identical to conventional cow's milk but does not require breeding and maintaining dairy cows for its production (Gasteratos 2019; Lawton 2021; Mendly‐Zambo et al. 2019). Scientists have estimated that the production of AFM emits 35%–96% less greenhouse gases and requires 77%–97% less land than the production of cow's milk (Lawton 2021; Mendly‐Zambo et al. 2019; Perfect Day Inc. 2021).

### 1.1 Perceptions of Milk Alternatives

Previous research has already identified some drivers of and barriers to consuming milk alternatives (e.g., Adamczyk et al. 2022; Giacalone et al. 2022; Haas et al. 2019; Pointke et al. 2022b). The most important reasons mentioned for consuming PBMAs include animal welfare, environmental protection, and the promotion of individual health (Adamczyk et al. 2022; Haas et al. 2019; Pointke et al. 2022b). Curiosity about and interest in new products also drive people to consume PBMAs and AFM (Adamczyk et al. 2022). In contrast, sensory properties (expected or real) and unfamiliarity with milk alternatives were identified as barriers. Accordingly, the acceptance of milk alternatives depends not only on the personal preferences of consumers but also on product‐related factors such as perceptions of milk alternatives (Giacalone et al. 2022).

In more detail, PBMAs are often perceived as less nutritious, more artificial, and more expensive than cow's milk (Haas et al. 2019; Kempen et al. 2017; Schiano et al. 2020). However, perceptions of PBMAs vary depending on the consumption of PBMAs. For example, consumers of PBMAs perceive them as natural, healthy, tasty, or nutritionally equivalent to cow's milk, while people who do not consume PBMAs perceive them as highly processed or artificial (Martínez‐Padilla et al. 2023). In addition, perceptions differ among the types of milk alternatives (Haas et al. 2019; Halme et al. 2023; Kempen et al. 2017). Oat milk, for example, is perceived as more environmentally friendly due to its lower carbon footprint than cow's milk (Halme et al. 2023).

Animal‐free dairy products such as cheese are often perceived as less tasty and natural than conventional cheese (Zollman Thomas and Bryant 2021). Zollman Thomas and Dillard (2022) also showed that consumers from the US, Singapore, the UK, and Germany perceived the production of animal‐free dairy products as technical and scary. However, animal‐free cheese was perceived as the most ethical and environmentally friendly product compared to conventional and vegan cheese in their study (Zollman Thomas and Bryant 2021).

Perceptions of milk alternatives are based on memorized or current product information (i.e., conceptions; Kroeber‐Riel and Gröppel‐Klein 2013). In our study, we define *conceptions* as mental constructs of varying complexity that concern a topic, object, or behavior, including ideas, beliefs, and knowledge (Duit et al. 2012; Gropengießer 2020). Conceptions can be categorized as cognitive processes, as cognitions include all forms of thinking and knowledge (American Psychological Association, 2022). Importantly, conceptions of an object or topic may be scientifically inaccurate or false (i.e., misconceptions), as conceptions may arise subconsciously (Coley and Tanner 2012). In consumer psychology, conceptions include subjective beliefs and knowledge about a product or product category based on individual experiences, social influences, and media information (Kroeber‐Riel and Gröppel‐Klein 2013). People already have simple conceptions about most products that can be deeply embedded. These conceptions affect how people perceive a product and thus influence consumer acceptance of that product (Kroeber‐Riel and Gröppel‐Klein 2013). For example, the perception of PBMAs as artificial might be based on complex conceptions about their production process. However, to the best of our knowledge, no study has yet examined young consumers' conceptions of PBMAs and AFM.

### 1.2 Aims of the Study

In this study, we aim to close this research gap by identifying and analyzing adolescents' conceptions of PBMAs and AFM. We focus on adolescents because younger people are more adventurous in their food choices, and their diets are more flexible than those of adults, which are important factors for shaping future dietary trends (Desbouys et al. 2021; Diethelm et al. 2012; Lytle et al. 2000; Shaikh et al. 2016). Furthermore, education can inform young people about how their diets affect human health, animal welfare, and the environment, prompting them to make responsible consumption choices. This can promote sustainable food consumption, as per the United Nations' *Sustainable Development Goals* (SDG 12; UNESCO 2017).

Thus, the first aim of our study was to examine adolescents' conceptions of milk alternatives—in particular, their conceptions of PBMAs and AFM, their ingredients, and production. This aim resulted in two research questions (RQs):

- RQ1: What are adolescents' conceptions of PBMAs, their ingredients, and their production?
- RQ2: What are adolescents' conceptions of AFM, its ingredients, and its production?

In Germany, people currently consume more cow's milk than PBMAs (Quantilope 2024), despite the consumption of milk in Germany decreasing in recent years (Bundesanstalt für Landwirtschaft und Ernährung 2024). Therefore, the second aim of our study was to analyze how the conceptions of adolescent cow's milk consumers (CMCs; i.e., non‐consumers of PBMAs), PBMA consumers (PBMACs), and consumers of both cow's milk and PBMAs (BCs) differ. A differentiated consideration of the conceptions of these user groups may enable the development of specific market strategies (e.g., by motivating casual consumers or CMCs to consume milk alternatives or by increasing the loyalty of existing PBMACs to milk alternatives; Martínez‐Padilla et al. 2023). This aim resulted in the third RQ:

- RQ3: How do conceptions of milk alternatives differ among adolescent CMCs, PBMACs, and BCs?

## 2 Milk Alternatives—Definition, Ingredients, and Production

### 2.1 PBMAs

PBMAs are defined as suspensions of water and dissolved plant components and are similar to cow's milk in their appearance, sensory properties, and possible uses (Bridges 2018; Mäkinen et al. 2016; Reyes‐Jurado et al. 2021). A general classification is made between PBMAs made from cereals (e.g., oats), legumes (e.g., soy), nuts (e.g., almonds), oilseeds (e.g., flaxseeds), and pseudo‐cereals (e.g., quinoa; Astolfi et al. 2020; Bridges 2018; Paul et al. 2019; Sethi et al. 2016).

PBMAs are produced by processing raw plant materials with water, turning them into a homogeneous liquid, following which stabilizers or other ingredients such as oil are added to achieve the desired texture and taste. In the first step, the plant's raw materials are soaked in water and then wet milled to extract nutrients such as carbohydrates, proteins, and fats (wet grinding). Alternatively, the plant's raw materials can be processed into flour and then mixed with water (dry grinding). The rough plant components are then separated from the resulting suspension via filtering, decanting, or centrifuging. Additional ingredients can then be added to formulate the product, including oils, salt, sugar, sweeteners, flavorings, or colorings, to improve consistency and/or taste. Emulsifiers and stabilizers may also be added to optimize the consistency and stability of the PBMA. This mixture of water, plant extracts, and other ingredients is then homogenized to produce a stable emulsion. After homogenization, the emulsion is heat‐treated via either pasteurization or ultra‐high temperatures. Both processes are used for preservation, resulting in the longer shelf life of PBMAs (Mäkinen et al. 2016; Reyes‐Jurado et al. 2021).

Due to the production process, PBMAs can be categorized as processed or ultra‐processed foods according to the NOVA food classification system (Monteiro et al. 2016). This categorization depends on the list of ingredients: PBMAs that consist of plant‐based raw materials, water, oil, and salt are considered processed foods, while those that contain emulsifiers or food colorings are considered ultra‐processed foods (Monteiro et al. 2019).

In the European Union, PBMAs may not be referred to as milk, as this term is legally protected (European Parliament and Council of the European Union 2013). Therefore, PBMAs are often marketed as a “drink” and not “milk” (e.g., oat drink) in Europe. In contrast, in the US, PBMAs are allowed to be sold and marketed as “milk”. The Food and Drug Administration (2023) recommends that producers indicate the nutritional values of PBMAs and their comparisons with those of conventional cow's milk on the packaging, though.

### 2.2 AFM

AFM is equivalent to cow's milk in appearance, sensory properties, and nutrients. The main ingredients are water and the cow milk proteins casein and whey protein, which are produced using precision fermentation. With this process, microorganisms (fungi or yeasts) are genetically modified and placed into a bioreactor with a culture medium to express casein and whey protein. These are then mixed with water, sugar, plant‐based fats, and minerals to produce animal‐free products such as AFM (Ercili and Barth 2021; Lawton 2021; Mendly‐Zambo et al. 2019; Waltz 2022).

In the US, Singapore, and Hong Kong, animal‐free dairy products made through precision fermentation have been available since 2021. In Europe, neither animal‐free dairy nor AFM is available, though. The Novel Food Regulation of the European Union is expected to approve AFM in Europe soon, as the ingredients of AFM (casein and whey protein) are produced with microorganisms (European Parliament and Council of the European Union 2015). In Germany, the food technology company *Formo* is working on the production of cheese alternatives using precision fermentation, and there were plans to introduce animal‐free cheese to the German market in 2024 (Formo 2022, 2024).

Based on the production process and the presence of casein and whey protein, AFM can be categorized as an ultra‐processed food according to the NOVA food classification system (Monteiro et al. 2019).

## 3 Methods

The materials and methods are described following the Qualitative Design Reporting Standards (JARS‐Qual; American Psychological Association 2018).

### 3.1 Study Design and Interview Procedure

We chose a qualitative approach using individual interviews and a semi‐structured interview guide to identify adolescents' conceptions of milk alternatives (Kumar 2024). A qualitative research design provides a detailed exploration of individual associations and conceptions that are difficult to access through standardized questionnaires (Flick 2018). In contrast, a semi‐structured interview guide provides flexibility, allowing participants to express their thoughts freely (Reinders 2016). The interview guide was tested in a pilot study with four adolescents before data collection to optimize the interview questions, prompts, and process (Döring and Bortz 2016). The pilot interviews were not included in the data analysis.

**FIGURE 1:**
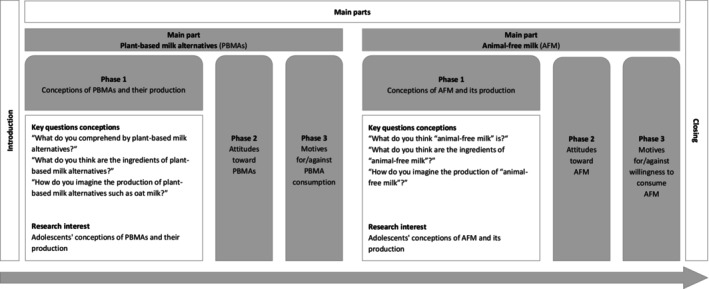
Key questions and research interests in the main phase of the interview regarding conceptions of milk alternatives. *Note*. Phases two and three of the main parts of the interview were not analyzed in this study. In the second phase, the adolescents were asked about their evaluation of plant‐based milk alternatives and animal‐free milk. In the third phase, the adolescents were asked about their motives for and against the consumption of plant‐based milk alternatives and animal‐free milk.

The interview guide was divided into four parts: (1) the introduction, (2) the main part concerning PBMAs, (3) the main part concerning AFM, and (4) the conclusion comprising a short questionnaire to characterize the participants (Figure 1). During data collection, the order of the two main interview parts was switched after each participant to minimize the possible influence of the order on participants' statements. The main parts were divided into three phases: (1) conceptions of PBMAs/AFM and their production, (2) attitudes toward PBMAs/AFM, and (3) motives for and against the consumption of PBMAs/the willingness to consume AFM (Figure 1). To address our RQs, only data from the first phase of the main parts were analyzed. The complete interview guide with the guiding questions and prompts is provided in Appendix S1.

In the first phase, the adolescents were asked about their conceptions of PBMAs/AFM, their ingredients, and their production. Participants were first asked what they understood by PBMAs/AFM. They were then asked about their conceptions of the ingredients of PBMAs/AFM. To capture adolescents' conceptions of PBMA production, they were first informed about PBMA ingredients by showing them an oat milk carton (water, oats [12%], sunflower oil, and sea salt). They were then asked how they imagined the production of a PBMA, such as oat milk, using these ingredients. As AFM is not yet available on the European market, it was not possible to present AFM and its ingredients. Thus, the adolescents were asked about their conceptions of producing AFM without further information and then received a short text about AFM for the second phase (Appendix S2). This text contained a brief explanation of the impact of livestock farming, what AFM is, and how it is produced. In the second phase, the adolescents were asked to evaluate PBMAs or AFM as “(rather) positive” or “(rather) negative” to assess their attitudes toward milk alternatives (Ajzen 2001). In the third phase, the adolescents were asked about their consumption of PBMAs or their willingness to consume AFM. Based on these results, the motives for and against the consumption of PBMAs/AFM were assessed with the questions “Why do you (never) drink PBMAs as an alternative to cow's milk?” and “Why are you (not) willing to consume AFM as an alternative to cow's milk?” (Kroeber‐Riel and Gröppel‐Klein 2013). This article focuses on the results from the first interview phase. The results of the second and third interview phases are presented in a seperate publication (Szczepanski et al. 2025). However, the motives for and against consuming PBMAs from the third interview phase were considered when describing the sample and discussing the results (Table 1).

**TABLE 1:** Overview of the sample (*N* = 25).

Name^a^	Gender	Age	Interview duration	Consumption cow milk^b^	Consumption PBMAs^c^	Willingness to consume AFM^d^	Motives for PBMA‐consumption^e^	Motives against PBMA‐consumption^f^
*Cows milk consumers*								
Liam	Male	17	50.30 min	Daily	Never	Willing	/	Lack of familiarity Social influence
Alexander	Male	17	47.63 min	Daily	Never	Not willing	Social norm	Price Taste and consistency
Mathys	Male	17	29.33 min	Daily	Never	Willing	Animal welfare	Social influence Convenience
Mila	Female	18	38.01 min	Daily	Never	Willing	/	Price Taste and consistency
Isabella	Female	18	50.67 min	Daily	Never	Willing	/	Taste and consistency Individual health
Amelia	Female	18	39.88 min	Daily	Never	Willing	/	Social influence/norm Convenience
Charlotte	Female	18	35.73 min	Daily	Never	Willing	/	Lack of familiarity Convenience
Maelys	Female	18	42.56 min	Weekly	Never	Willing	Animal welfare Environmental protection	Taste and consistency
*Plant‐based milk alternative consumers*								
Oliver	Male	17	41.40 min	Never	Weekly	Willing	Individual health Social influence	/
Samuel	Male	18	46.07 min	Never	Weekly	Willing	Taste and consistency Environmental protection	Environmental protection
Sophie	Female	18	40.47 min	Never	Weekly	Willing	Animal welfare Social influence/norm	/
Emmy	Female	17	46.07 min	Never	Weekly	Willing	Individual health Animal welfare	Uncertainties
*Cows milk and plant‐based milk alternative consumers*								
Jack	Male	18	34.73 min	Daily	Daily	Willing	Environmental protection Individual health	Taste and consistency
Lucas	Male	18	37.63 min	Daily	Daily	Willing	Social influence Environmental protection	/
Mason	Male	17	46.80 min	Weekly	Daily	Willing	Environmental protection Individual health	Price
Lily	Female	18	35.07 min	Weekly	Daily	Willing	Individual health Animal welfare	Price Taste and consistency
Emma	Female	17	45.11 min	Monthly	Daily	Willing	Individual health Animal welfare	Environmental protection
Mia	Female	19	49.21 min	Monthly	Daily	Willing	Environmental protection	/
Ella	Female	18	51.63 min	Weekly	Weekly	Willing	Environmental protection Animal welfare	Social norm Taste and consistency
Olivia	Female	18	47.87 min	Weekly	Weekly	Willing	Environmental protection Animal welfare	Lack of familiarity Taste and consistency
Emily	Female	18	35.78 min	Weekly	Weekly	Willing	Taste and consistency Individual health	Social influence/norm Taste and consistency
Benjamin	Male	16	41.13 min	Weekly	Weekly	Willing	Individual health Animal welfare	/
Emilia	Female	17	32.32 min	Monthly	Weekly	Willing	Environmental protection Individual health	Social influence
Sophia	Female	17	43.11 min	Weekly	Monthly	Willing	Animal welfare Environmental protection	Taste and consistency
Elias	Male	18	39.01 min	Monthly	Monthly	Not willing	Generation ideales Environmental protection	Price

*Note:* “/” means that no motives for or against consuming PBMAs were given.

Abbreviations: AFM, animal‐free milk; PBMAs, plant‐based milk alternatives.

^a^ Randomly selected pseudonym according to the most popular first names in 2022 in the United States of America.

^b^ The adolescents were asked about their consumption of cow's milk after the interview and could choose daily, weekly, monthly, or never.

^c^ The adolescents were asked about their consumption of PBMAs after the interview and could choose daily, weekly, monthly, or never.

^d^ During the interview, the adolescents received an informative text about AFM and were asked to decide accordingly whether they were willing or unwilling to consume AFM.

^e^ In the third phase of the interview, the adolescents were asked what they considered to be the most important motive for the consumption of PBMAs.

^f^ In the third phase of the interview, the adolescents were asked what they considered to be the most important motive against the consumption of PBMAs.

At the end of the interview, a short questionnaire was completed by each participant that included sociodemographic information and questions about their consumption of cow's milk and milk alternatives (Appendix S1). The questionnaire was purely descriptive (not a psychometric measuring instrument). It was used to collect data on gender (male, female, or other), place of residence (urban or rural), diet (omnivorous, vegetarian, vegan, or other), and consumption of plant‐based drinks (never, monthly, weekly, or daily) and cow's milk (never, monthly, weekly, or daily; Table 1).

### 3.2 Sample and Data Collection

For sample construction, the inductive sampling method for exploratory studies, according to Reinders (2016), was chosen. The sample was selected based on three criteria: participants had to (1) be at least upper‐secondary school students, (2) identify with different genders, and (3) consume either cow's milk or PBMAs. The criteria ‘upper‐secondary school’ ensured that the adolescents had sufficient biological knowledge to understand the production of AFM for the interview phases two and three. The criteria “gender” and “consumer behavior” were chosen to determine the conceptions, attitudes, and motives of adolescents of all genders and consumer groups.

In September and October 2022, we interviewed 25 adolescents from Germany aged between 16 and 19 (*N* = 25; *M*_Age_ = 17.60 years; *SD*_Age_ = 0.63 years; 60% female). The interviews took an average of 41.86 min (*SD*_Duration_ = 6.07 min). The adolescents came from five secondary schools in the city and district of Osnabrück (Lower Saxony, Germany). They were in the eleventh to thirteenth grades (upper‐secondary school) and living with their parents at the time of data collection, with 13 living in a rural and 12 in an urban area. Fifteen adolescents were omnivorous, seven were vegetarian, two were flexitarian, and one was vegan. Eight adolescents reported not consuming PBMAs but consuming cow's milk daily or weekly. Four adolescents reported not consuming cow's milk but consuming PBMAs weekly. Thirteen adolescents consumed both cow's milk and PBMAs. The most important reasons for the consumption of PBMAs for the adolescents in our study were environmental protection, animal welfare, and health promotion. The main reasons against the consumption of PBMAs were taste and consistency, social influence, social norms, and price (Table 1). Descriptive statistics of the sample and their self‐reported consumption of cow's milk, PBMAs, and their willingness to consume AFM are presented in Table 1. All names mentioned in the article have been replaced with pseudonyms to ensure anonymity.

School principals of all secondary schools in Osnabrück were contacted via email to recruit upper‐secondary school students to participate in the study. The email contained a description of the research project and a request for permission to collect data from upper‐secondary school students. The ethics approval for the study by the Regional State Office for Schools and Education Osnabrück (OS 1 R.24–0541 2/N) was also attached. Principals forwarded the invitation to biology teachers, who then recruited voluntary participants. The biology teachers were instructed to ensure an equal distribution of genders and consumption of PBMAs and cow's milk when selecting participants. The interviews were scheduled in blocks, with participants from the same school being interviewed on the same day. After each interview block, content saturation was discussed by two coders. The two coders used the deductive category system as a foundation, in addition to the audio recordings, to discuss whether new content and inductive codes had arisen. After 21 interviews, the content was considered to be saturated. At this point, four more interviews were scheduled, which were then conducted. This resulted in a final sample size of 25.

Consent declarations were signed by the school principals, the students, and—in the case of underage students—the parents before the data were collected. The students and parents also received an information letter informing them about the aims of the study, the voluntary nature of participation, and the anonymization of the data. The content of the interview guide was not communicated to the students before the study. Participation was voluntary and anonymous, and students could withdraw from the study without consequences.

### 3.3 Data Processing and Analysis

Interview audio records were transcribed according to Dresing and Pehl (2018). The transcripts were then redacted according to Gropengießer (2008) to improve readability and reduce the content in the transcripts concerning the research questions. The transcription and redaction rules are provided in Appendix S3. The redacted data were analyzed in MAXQDA 2020 (Verbi. 2020) using qualitative content analysis (Kuckartz and Rädiker 2019). This was done to compare the respondents' conceptions with the scientific content. As our sample was not representative of adolescents in Germany, we did not aim to make any generalized statements; however, we used frequency data to illustrate trends within our sample (American Psychological Association 2018).

Based on the research questions and interview phases, a deductive category system was developed with the following superordinate categories: “Conceptions of PBMAs” and “Conceptions of AFM.” The two superordinate categories were each subdivided deductively into three subcategories, “Definition PBMAs/AFM,” “Ingredients of PBMAs/AFM,” and “Production of PBMAs/AFM”. Within each subcategory, deductive codes were created based on the science content concerning PBMAs/AFM, their ingredients, and the production process (Chapters 2.1 and 2.2). Inductive codes (*) were derived from the statements made by the participants during data analysis (Tables 2 and 3).

**TABLE 2:** Category system for analyzing adolescents' conceptions of PBMAs.

Conceptions of PBMAs		
Definition PBMAs	Ingredients PBMAs	Production of PBMAs
Aloe vera milk	Additives*	Cleaning raw plant material*
Banana milk*	Binders*	Cultivation/Harverst raw plant material*
Cereal milk	Chemicals*	Dry or wet grinding
Coconut milk*	Emulsifiers*	Filtration
Cow's milk alternative	Fat*	Heat treatment
Legume milk	Fiber*	Homogenization
Nut milk	Flavor enhancer*	Mixing ingredients*
Oilseed milk	Flavors*	Packaging
Smoothie*	Food colorings*	Product formulation
	Minerals	Pressing raw plant material*
	Nutrients*	Quality tests/controls*
	Oil	Soaking/Mixing with water
	(Plant‐based) proteins*	
	Preservatives*	
	Raw plant material	
	Salt	
	Stabilizers	
	Sweeteners/Sugar	
	Vitamins	
	Water	

Abbreviation: PBMAs, plant‐based milk alternatives.

* Inductively coded categories.

**TABLE 3:** Category system for analyzing adolescents' conceptions of AFM.

Conceptions of AFM		
Definition AFM	Ingredients AFM	Production of AFM
Animal‐friendly milk*	Additives*	Copying milk*
Artificial milk*	Binders*	Cultivating milk cells*
Cow's milk equivalent	Cereals*	(Further) processing
Lab milk*	Chemicals*	Heat treatment*
Plant‐based/Animal‐free*	Different substances*	Modification of microorganisms
	Fat*	Multiplication of milk glands*
	Flavor enhancer*	Precision fermentation
	Flavors*	Production in a bioreactor*
	Food supplements*	Production with bacteria*
	Lactose*	Production with genetic engineering*
	Milk cells*	Production with yeast*
	Milk proteins	Recycling of old dairy products*
	Minerals	
	Nuts*	
	Oats*	
	Oil*	
	Plant‐based fats*	
	Plant substances*	
	Salt*	
	Soy*	
	Sugar*	
	Water	

Abbreviation: AFM, animal‐free milk.

* Inductively coded categories.

To compare the conceptions of adolescents who consumed cow's milk, PBMAs, or both, participants were assigned to one of three consumption groups (CMCs, *n* = 8; PBMACs, *n* = 4; and BCs, *n* = 13) based on their self‐reported consumption habits (RQ3; Table 1). The complete category system can be found in Appendix S4.

To confirm the internal coding agreement, 20% of all relevant statements that answered the RQs were additionally coded by the second author (Kuckartz and Rädiker 2019). To assess the agreement between the coders, the kappa coefficient, according to Brennan and Prediger (1981), was calculated in MAXQDA. The resulting kappa value was 0.80, indicating near‐perfect intercoder agreement (Landis and Koch 1977).

## 4 Results

### 4.1 Conceptions of PBMAs

#### 4.1.1 Definition of PBMAs

All adolescents in our study were familiar with at least one PBMA, with cereal‐based (*n* = 29), nut‐based (*n* = 27), and legume‐based (*n* = 23) PBMAs being the most mentioned. Oat milk (*n* = 23) was the best‐known PBMA, followed by soy (*n* = 21) and almond (*n* = 21).

Most adolescents perceived PBMAs as a substitute for cow's milk (*n* = 16), with Sophie, Mila, and Charlotte emphasizing the role of PBMAs for people with a vegan diet or lactose intolerance (Sophie: “PBMAs are definitely something that you can consume as a substitute for milk […] or if you are lactose intolerant”). Only Emma, Charlotte, and Lucas provided more details; specifically, that PBMAs are a suspension of water and plant components that has a “[…] milk‐like taste, consistency and color […]” (Charlotte). Furthermore, all adolescents in our study referred to PBMAs as milk (e.g., oat milk) and not as a drink.

#### 4.1.2 Ingredients and the Production of PBMAs

Almost all the adolescents in our study named water (*n* = 25) and raw plant material such as oats (*n* = 23) as the main components of PBMAs (e.g., Isabella: “Oats or almonds are naturally in it and water, of course, to make it liquid”); however, the participants had different ideas about the proportion of the two main components. For some, they believed the main ingredient was the raw plant material, which is mixed with “[…] a little bit of water […]” (Lucas) to produce the liquid. Others believed water was the “[…] basic substance of plant‐based milk […]” (Amelia). The adolescents thought the third component of plant milk was “[…] probably sugar/sweetener” (*n* = 14). Other ingredients of PBMAs such as oil (*n* = 0), salt (*n* = 2), minerals (*n* = 4), vitamins (*n* = 1), and stabilizers (*n* = 0) were rarely mentioned (Table 4).

**TABLE 4:** Overview of adolescents' conceptions of PBMAs and their production.

Ingredients of PBMAs		Production of PBMAs	
Category (Example^b^)	Frequency, *n* (%)^a^	Category (Example^b^)	Frequency, *n* (%)^c^
Water	25 (27.5)	Soaking/Mixing with water (soaked in or mixing the raw plant material with water)	19 (22.6)
Raw plant material (oat)	23 (25.3)	Dry or wet grinding (crushing of the raw plant material)	16 (19.0)
Sweeteners/Sugar	14 (15.4)	Mixing ingredients*	15 (17.9)
(Plant‐based) proteins*	4 (4.4)	Heat treatment (heating the water‐plant‐mixture)	10 (11.9)
Minerals (Calcium)	4 (4.4)	Filtration (sieving or filtering the water‐plant‐mixture)	7 (8.3)
Additives*	4 (4.4)	Pressing raw plant material*	6 (7.1)
Flavor enhancer*	3 (3.3)	Product formulation (product customization by adding further ingredients)	4 (4.8)
Preservatives*	2 (2.2)	Cultivation raw plant material*	3 (3.6)
Salt	2 (2.2)	Packaging	2 (2.4)
Flavors*	1 (1.1)	Cleaning raw plant material*	1 (1.2)
Fiber*	1 (1.1)	Quality tests/controls (testing the quality and safety of the product)*	1 (1.2)
Binders*	1 (1.1)	Homogenization	0
Chemicals*	1 (1.1)		
Emulsifiers*	1 (1.1)		
Food colorings*	1 (1.1)		
Fat*	1 (1.1)		
Nutrients*	1 (1.1)		
Stabilizers	1 (1.1)		
Vitamins (e.g., Vitamin B12)	1 (1.1)		
Oil (e.g., sunflower oil)	0		

Abbreviation: PBMAs, plant‐based milk alternatives.

* Inductively coded categories.

^a^ Percentage of all coded segments in the category “Ingredients plant‐based milk alternatives” (*N* = 91).

^b^ The examples are based on the science content for the deductive codes and on the participants' statements for the inductive codes.

^c^ Percentage of all coded segments in the category “Production plant‐based milk alternatives” (*N* = 84).

Regarding the production process of PBMAs, most study participants envisioned two steps needed to produce oat milk: soaking or mixing raw plant material with water (*n* = 19) and dry or wet grinding of raw plant material (*n* = 16) (Table 4). Emma described the production process: “First, the oats or almonds are soaked in water, and then everything is mixed to get a smooth liquid.” Participants predominantly shared the idea that the ingredients (water and oats) were mixed, and then “[…] oat milk is produced by stirring” (Mathys). Olivia's description provides insights into her view of the production process: “I think water is the basis of oat milk. Then, I guess water is mixed with oats, and probably the rest, like salt and oil, is added. I would say it's all mixed.” Subsequently, only some participants envisioned how the oat‐water mixture would be further processed. They described that, after mixing the oats and water, the mixture is filtered (*n* = 6), and eventually, “[…] something happens with heat” (Mila) (*n* = 10). Mila stated: “You mix the ingredients in a pot and heat them up. Then oat milk comes out.” Only Benjamin explained the purpose of heat treatment in this context: “[…] PBMAs have to be heated so that they last longer, as with pasteurization.” None of the adolescents imagined that technical processes such as homogenization would be carried out on PBMAs to achieve the required consistency and shelf life. Furthermore, some adolescents expressed uncertainties about the production process and said that they either knew little about the process or had not thought about it. For example, Emma mentioned: “I've never actually thought about how exactly plant milk is made.” Isabella agreed, stating: “It's something I've never really thought about.”

### 4.2 Conceptions of AFM

#### 4.2.1 Definition of AFM

Nearly all adolescents in our study conceptualized AFM as a vegan/PBMA (*n* = 24), which “[…] is free from animal ingredients and no animals are involved in its production […] ” (Lucas). They had difficulty differentiating AFM from PBMAs, often perceiving both as animal‐free products. For example, Mila stated:

I imagine that AFM is something that doesn't contain any animal ingredients, so it's not going to be regular milk. I just don't know what the difference is to PBMAs because they don't contain any animal ingredients either.

After the adolescents expressed their conceptions of AFM, they were informed that AFM is not a PBMA. As a result, some imagined AFM as an artificial (*n* = 9) or laboratory milk (*n* = 7). However, the participants then stated that they did not have differentiated conceptions of artificial or laboratory milk, as Amelia and Charlotte explained:

Well, if it's not plant‐based and not animal‐based, then it can only be chemical and artificial. But I don't have any idea what it's like. (Amelia)

If it's not plant‐based, it sounds very artificial to me, a bit synthesized. (Charlotte)

#### 4.2.2 Ingredients and the Production of AFM

Most of our study participants had limited or no conceptions of the ingredients and production of AFM (Table 5). Lily, Benjamin, Emmy, and Mathys stated:

I have no idea what is in animal‐free milk because you only know the plant‐based milk alternatives. (Lily)

I have no idea what is in this artificial milk product. (Benjamin)

I couldn't imagine what it is made of. (Emmy)

I think this milk alternative consists of substances or materials with similar flavors to those in regular milk, but I can't imagine what that could be. (Mathys)

Some adolescents imagined that AFM contains water, as with PBMAs (*n* = 7). A few speculated about other ingredients, such as salt (*n* = 5), chemicals (*n* = 4), flavor enhancers (*n* = 4), minerals (4), and plant substances (*n* = 4). For example:

There will be water in it and probably salt. I can't think of anything else off the top of my head. (Liam)

I imagine that animal‐free milk contains water and salt. Salt is in everything. For the rest, I have no idea. (Mila)

While most participants had no conception of the ingredients of AFM, a few attempted to deduce potential ingredients from their conception as artificial or laboratory milk. They imagined that AFM consisted of chemicals or flavor enhancers. None imagined that milk proteins are the basic component of AFM (Table 5).

**TABLE 5:** Overview of adolescents' conceptions of AFM and its production.

Ingredients of AFM		Production of AFM	
Category (Example^b^)	Frequency, *n* (%)^a^	Category (Example^b^)	Frequency, *n* (%)^c^
Water	7 (14.0)	Production with bacteria*	1 (11.1)
Salt*	5 (10.0)	Production in a bioreactor*	1 (11.1)
Chemicals*	4 (8.0)	Production with genetic engineering*	1 (11.1)
Flavor enhancer*	4 (8.0)	Production with yeast*	1 (11.1)
Minerals (calcium)	4 (8.0)	Heat treatment*	1 (11.1)
Plant substances*	4 (8.0)	Copying milk*	1 (11.1)
Lactose*	3 (6.0)	Recycling of old dairy products*	1 (11.1)
Nuts (almonds)*	3 (6.0)	Multiplication of milk glands (Multiplication of milk glands in the lab to produce milk)*	1 (11.1)
Soy*	3 (6.0)	Cultivating milk cells*	1 (11.1)
Oat*	2 (4.0)	Precision fermentation	0
Oil*	2 (4.0)	Modification of microorganisms (Modification of the genetic information)	0
Flavors*	1 (2.0)	(Further) processing	0
Binders*	1 (2.0)		
Fat*	1 (2.0)		
Cereals*	1 (2.0)		
Milk cells*	1 (2.0)		
Food supplements*	1 (2.0)		
Different substances*	1 (2.0)		
Sugar*	1 (2.0)		
Additives*	1 (2.0)		
Milk proteins	0		
Plant‐based fats	0		

Abbreviation: AFM, animal‐free milk.

* Inductively coded categories.

^a^ Percentage of all coded segments in the category “Ingredients animal‐free milk” (*N* = 50).

^b^ The definition is based on the science content for the deductive codes and on the participants' statements for the inductive codes.

^c^ Percentage of all coded segments in the category “Production animal‐free milk” (*N* = 9).

The adolescents' conceptions of AFM production were also limited, with the majority having no conceptions about how AFM is produced (Table 5). A few imagined that “cow's milk is copied to artificially produce AFM” (Benjamin). More specifically, Emilia, Olivia, and Liam stated that microorganisms such as yeasts or bacteria and a bioreactor are needed for producing AFM. Although the adolescents had only vague conceptions of AFM production, they imagined the process to be complex as the product “[…] contains more chemicals” (Mila). Mila, Ella, and Jack imagined, based on their subjective knowledge of in cultivated meat production, that milk‐producing tissue would be cloned in the lab to create AFM. They had the following conceptions:

I could even imagine that genetic engineering was used somehow, that maybe cells were copied, and animal clones were created. (Ella)

Then it would be like cultivated meat but in the form of milk. […] Perhaps you could use mammary glands to produce AFM so that you could multiply them and then, I don't know, whether it could work to make them produce milk. (Mia)

### 4.3 Conceptions of CMCs, PBMACs, and BCs

#### 4.3.1 Comparison of Consumer Groups' Conceptions of PBMAs

The CMCs in our study were less familiar with PBMAs than the PBMACs and BCs. For example, the CMCs were only aware of oat, almond, and soy milk as PBMAs, while the PBMACs and BCs mentioned a variety of PBMAs, including cashew, walnut, hazelnut, hempseed, rice, spelt, and pea milk. In addition, the CMCs classified PBMAs as a substitute for lactose‐intolerant individuals and vegans, while the PBMACs and BCs regarded it as a cow's milk alternative. Mila (a CMC) described PBMAs as “products that are supposed to replace milk […] for lactose‐intolerant people or […] for vegans.” For the CMCs, PBMACs, and BCs, water, raw plant materials, and sugar were believed to be the basic ingredients of PBMAs, while CMCs thought that PBMAs also contained additives, binders, or flavor enhancers. PBMACs and BCs mentioned micronutrients, primarily calcium, as further ingredients. The CMCs imagined that PBMAs were mainly produced in two steps: grinding the plant's raw material and mixing it with water. Charlotte described it as follows: “I guess the oats are ground to a very powdery state […] then I would add water to them.” Only Mila and Alexander assumed, as a parallel to the production process of cow's milk, that “something with heat […]” happens at the end (Mila). The PBMACs and BCs held more differentiated conceptions of PBMA production. The PBMACs imagined the production of PBMAs in four steps: “The oats are soaked in water” (Sophie), and then “[…] everything is mixed, and the solids are separated from the liquids” (Samuel). Then, “[…] everything is heated again” (Oliver). The BCs also described the grinding of the plant's raw material, mixing with water, and heating as the basic steps in the production of PBMAs (Mason, Lily, and Emily). In addition, the BCs also provided reasons for the individual production steps:

I think it's filtered […] because you still have oat solids that don't belong in it. Sunflower oil and salt are then added. […] If I remember correctly, salt also helps to preserve things. (Mason)

The sunflower oil is […] added at the end for consistency. (Lily)

#### 4.3.2 Comparison of Consumer Groups' Conceptions of AFM

CMCs, PBMACs, and BCs held similar ideas about AFM. After being informed that AFM is not a PBMA, they were unable to conceive of anything else as an alternative to milk, or they categorized AFM as laboratory, artificial, or synthetic milk. The PBMACs had few ideas about the ingredients and production of AFM, and only Sophie assumed that “[…] the combination [of] […] different chemical substances create a milky consistency that you can also drink” (Sophie). The CMCs imagined water as the basis for producing AFM. In their conception, the water was mixed with different (chemical) substances, flavor enhancers, additives, and binders. The BCs also mentioned water as the basic ingredient of AFM, which was mixed with plant‐based substances, minerals, and lactose. Jack was the only one who imagined milk cells were the basis for producing AFM. Regarding AFM production, some of the BCs (Benjamin, Emilia, Mia, Jack, Olivia, and Ella) had their first ideas about the synthetic/artificial production of AFM. Jack imagined that milk cells could be grown in a laboratory with the same structure and taste as milk. Benjamin, in contrast, considered whether milk could be copied in a laboratory.

## 5 Discussion

### 5.1 Key Findings and Their Interpretation

#### 5.1.1 Conceptions of PBMAs

Our results indicate that most adolescents in our study were familiar with PBMAs as an alternative to cow's milk, with oat, soy, and almond milk being the best‐known alternatives (POSpulse GmbH 2021). This may be explained by the growing range of PBMAs available in supermarkets and cafés. *REWE*, Germany's second‐largest food retailer, currently offers six types of PBMAs, including oat, soy, almond, coconut, rice, and pea milk (REWE 2024). In addition, the Nutrition Report from Germany has shown that the consumption of vegan alternatives to animal products has increased since 2020, especially among young people aged 14–29 years (Deutsche Gesellschaft für Ernährung 2024). For example, 18% of all young people in Germany reported consuming meat and milk alternatives daily or several times a day (Federal Ministry of Food and Agriculture 2024). Furthermore, the statements of the adolescents in this study indicate that they accept PBMAs as a vegan substitute for milk, as they used the term' milk' when referring to PBMAs (e.g., oat milk). However, it should be noted that only a product of udder secretion obtained by milking may be called milk in Europe (European Parliament and Council of the European Union 2013). They also associated the use of PBMAs with specific diets, such as vegan or lactose‐free. One reason could be that the number of young people in Germany who follow a lactose‐free diet has increased, as 29% of all lactose‐intolerant individuals in Germany are between 14 and 29 years old (Statista 2024).

The adolescents in our study held a basic but simplified understanding of the ingredients of PBMAs. In addition to the basic ingredients (water, oats, and sugar), they expressed uncertainties regarding other ingredients, indicating a knowledge gap. As Isabella stated, “Besides oats, water, and sweetener, PBMAs contain other ingredients, this is always the case in the industry. But I don't know exactly what could also be in PBMAs.” This knowledge gap specifically relates to ingredients not directly recognizable in PBMAs, such as oil, stabilizers, micronutrients, and vitamins.

Adolescents' conceptions may be based on their product perceptions. For example, the adolescents in our study mentioned sugar or sweeteners as one main ingredient of PBMAs, even though no additional sugar is added to many of them (Pointke et al. 2022a; Singh‐Povel et al. 2022). However, oat milk, which is the most consumed PBMA in Germany (Statista 2021, 2024), is sweeter than cow's milk due to its higher carbohydrate content (Paul et al. 2019; Singh‐Povel et al. 2022). Adolescents' conception that sugar or sweeteners might be an ingredient could have arisen more from sensory experiences than specific nutritional knowledge. This assumption is supported by the fact that (young) people's food choices are based on the interplay of social and biological factors such as sociability, price, and visual attractiveness, and not specifically based on knowledge about the food (Markovina et al. 2015; Renner et al. 2012).

In addition, the adolescents in our study held simplified conceptions regarding the production process of PBMAs. They regarded their production as uncomplicated or simplified (i.e., grind oats, mix with water, and heat the liquid if necessary) and openly admitted their uncertainties or knowledge gaps. This is consistent with their limited familiarity with the ingredients of PBMAs. This lack of awareness could be influenced by the product marketing of PBMAs and a lack of nutritional knowledge. Popular PBMA brands such as *Alpro* and *Oatly* focus on marketing PBMAs as a natural and sustainable alternative to cow's milk without explaining their production (Alpro 2024; Oatly 2024). Furthermore, nutrition education at secondary schools in Germany focuses on national nutrition recommendations, dietary guidelines and nutrients and their function. Consumer education regarding the production of food or food labeling is not firmly anchored in the national curriculum. Nutritional education and teaching it in the classroom is also the responsibility of teachers. Thus, no conclusion can be drawn about its inclusion in teaching (Heseker et al. 2019).

#### 5.1.2 Conceptions of AFM

The adolescents in our study held undifferentiated and vague conceptions of AFM. Almost all study participants incorrectly imagined AFM to be vegan/PBMAs. They constructed this misconception from the term “animal‐free.” Consequently, participants were unable to distinguish AFM from PBMA as they were unfamiliar with the term animal‐free for precision‐fermented dairy products. Although previous studies have shown that labeling precision‐fermented dairy products as animal‐free dairy has a positive effect on potential consumers in Germany (The Good Food Institute 2024a), our results suggest that using the term without further information can lead to uncertainties and confusion. This lack of familiarity was also reflected in conceptions such as laboratory milk. The adolescents derived their conceptions of AFM as artificial, synthetic, or laboratory milk from cultivated meat, which is still described in the media with phrases such as “grown in the laboratory” (The Good Food Institute 2024b). Emma stated: “Maybe it's something that's produced in a laboratory, like cultivated meat,” while Mia speculated that “It sounds similar to cultivated meat, only in milk form.” However, it should be noted that the production technologies for cultivated meat (cell cultivation) and AFM (precision fermentation) differ.

The adolescents' lack of familiarity can be explained by the fact that precision fermentation‐based milk alternatives are not yet available on the European market, and (young) people are not familiar with novel food technologies. However, the number of fermentation‐based companies in Europe is growing, and these companies are attempting to address regulatory issues and familiarize consumers with fermentation technologies (The Good Food Institute 2024a).

Most of the adolescents in our study had no or vague (sometimes even false) conceptions of AFM and its production. Despite existing uncertainties and a lack of familiarity, participants perceived the AFM production process as complex and synthetic. This perception appears to have been influenced by the conceptions of AFM as lab‐grown or artificial, which, in turn, stems from the naming of cultivated meat as lab‐grown meat (The Good Food Institute 2024b).

Although some adolescents mentioned genetic engineering, microorganisms, or bioreactors, none were familiar with precision fermentation. People in the US are also less familiar with–and skeptical about–precision fermentation (Hartman Group 2023). However, the Hartman Group (2023) demonstrated that a brief description of precision fermentation technology positively affects people's willingness to purchase these products. This could also prevent misconceptions. In our study, three adolescents had the misconception that milk‐producing tissue is copied in the laboratory to produce AFM, which was based on existing uncertainties about the production process and new food technologies.

#### 5.1.3 Comparison of Consumer Groups' Conceptions of PBMAs and AFM

The adolescents' conceptions of PBMAs differed depending on their cow milk and PBMA consumption. CMCs had limited familiarity with PBMAs and were familiar only with common PBMAs such as oat, almond, or soy milk. They associated PBMAs primarily with specific diets such as vegan or lactose‐free. CMCs also held basic conceptions regarding the ingredients and production of PBMAs, whereby they thought of additives, binders, or flavor enhancers as additional ingredients. From this, it can be assumed that young people who consume cow's milk perceive PBMAs as less natural or more processed than cow's milk. In contrast, PBMACs and BCs were familiar with PBMAs and their possible applications. Their conceptions included more detailed production processes such as soaking, filtering, and heating, and they considered micronutrients such as calcium as ingredients. The PBMACs and BCs consumed PBMAs for environmental protection, animal welfare, and promotion of their health, which may explain their more differentiated conception of PBMAs.

The adolescents' conceptions of AFM were similar, limited, and independent of their cow's milk and PBMA consumption. Most adolescents categorized AFM as laboratory milk, artificial milk, or synthetic milk produced using water and chemicals. The adolescents held few ideas concerning how AFM is produced, and only the BCs speculated about laboratory‐based methods, such as the cultivation of milk cells.

Overall, our results indicate that young people are unfamiliar with milk alternatives based on precision fermentation and perceive them as artificial. This perception may be based on the novelty of the product, which is not yet available on the European market.

## 6 Limitations

As we used a qualitative interview approach with specific characteristics for the sample, we did not have a representative sample of adolescents in Germany. Based on our findings, it is impossible to make generalized statements regarding adolescents' conceptions of PBMAs and AFM. Furthermore, all adolescents in this study had a high level of education (upper‐secondary level), and previous studies have shown that people with a high level of education have a higher interest in more sustainable diets (Mensink et al. 2016; Zhu et al. 2013).

Cow's milk is a staple food in Germany and, according to the German Nutrition Society, is considered part of a healthy diet (Deutsche Gesellschaft für Ernährung 2021, 2024). The status of cow's milk in German society may inhibit interest in milk alternatives (see also motives against the consumption of PBMAs, Table 1). Therefore, the individual interests, educational level, and cultural background of the adolescents in our study should be taken into consideration when interpreting our results.

Furthermore, labeling milk produced via precision fermentation as AFM represents a methodological limitation. Zollman Thomas and Dillard (2022) demonstrated that different names for dairy products produced with precision fermentation affect potential consumers differently. Following Zollman Thomas and Dillard's (2022) recommendations, we used the term AFM because it is simple and differs from cow's milk. Nevertheless, the use of the English term AFM confused the adolescents during the interviews as they often assumed that it referred to PBMAs. Despite these limitations, we gained insight into young people's conceptions of already established (PBMAs) and future milk alternatives (AFM).

## 7 Implications for Research and Education

Implications for future research and education are based on the findings that (1) adolescents have limited conceptions of the ingredients and production of PBMAs and (2) have little or no conceptions of AFM.

Young people should be prepared to make individual decisions on controversial topics such as the consumption of animal‐based versus plant‐based foods (Monaco Bissonnette and Contento 2001). Food choices are a daily behaviors that involve economic, social, and health concerns (Von Koerber et al. 2017). Therefore, the consumption of cow's milk (as a norm in Germany) or milk alternatives should be addressed in schools as part of the United Nations *Education for Sustainable Development* to enable young people to position themselves for responsible consumption when making food choices (Sustainable Development Goal 12) (UNESCO 2017). Therefore, young people should be informed about cow's milk and milk alternatives as a part of their nutritional education, with ingredients and production processes providing the basis for evaluating their advantages and disadvantages (Monaco Bissonnette and Contento 2001). Martínez‐Padilla et al. (2023) and Broad et al. (2022) also pointed out that the provision of information on milk alternatives (PBMAs and AFM) is necessary to improve individuals' understanding and perception of these products. In particular, AFM should be introduced to (young) people before its market launch, as the adolescents in our study imagined AFM to be laboratory, synthetic, or artificial milk due to a lack of information. The Hartman Group (2022) was able to show that a description of the ingredients and the production of food based on precision fermentation increases the willingness to buy. However, before addressing the consumption of cow's milk and milk alternatives in class, teachers should discuss the need to restructure current dietary habits with their students, given the different attitudes that determine young people's behavior (Swaim et al. 2014).

Food choices are more intuitive than rational (Köster 2009). Therefore, nutritional education should aim to encourage young people to critically question current dietary habits. As the adolescents in our study had limited conceptions of milk alternatives, nutritional education should also include how to deal with uncertainties in food choices, especially as young people are most likely to use packaging labels and social media as sources of information (Martínez‐Padilla et al. 2023; Monaco Bissonnette and Contento 2001).

Future research should examine the role of conceptions and uncertainties regarding cow's milk and milk alternatives on young people's food choices. Furthermore, the role of conceptions in the evaluation of cow's milk and milk alternatives should be investigated.

## Author Contributions

**Lena Szczepanski:** conceptualization (lead), data curation (lead), formal analysis (lead), investigation (lead), methodology (lead), project administration (equal), writing – original draft (lead), writing – review and editing (lead). **Insa Stonner:** formal analysis (supporting), writing – original draft (supporting). **Florian Fiebelkorn:** conceptualization (supporting), project administration (equal), resources (lead), supervision (lead), writing – review and editing (supporting).

## Ethics Statement

All subjects gave informed consent for inclusion before participating in this study. The study was carried out following national and institutional guidelines, the Declaration of Helsinki, the German Research Foundation, and the American Psychological Association (American Psychological Association 2017; Deutsche Forschungsgemeinschaft e.V. (DFG) 2022; World Medical Association 2022). Ethics approval for the present study was issued by the Regional State Office for Schools and Education Osnabrück (OS 1 R.24–0541 2/N).

## Conflicts of Interest

The authors declare no conflicts of interest.

## Supporting information


Appendix S1.

Appendix S2.

Appendix S3.

Appendix S4.

## Data Availability

The data supporting the conclusions of this article are available from the corresponding author upon reasonable request.

## References

[fsn370259-bib-0001] Adamczyk, D. , D. Jaworska , D. Affeltowicz , and D. Maison . 2022. “Plant‐Based Dairy Alternatives: Consumers' Perceptions, Motivations, and Barriers—Results From a Qualitative Study in Poland, Germany, and France.” Nutrients 14, no. 10: 2171. 10.3390/nu14102171. 35631311 PMC9147774

[fsn370259-bib-0002] Ajzen, I. 2001. “Nature and Operation of Attitudes.” Annual Review of Psychology 52: 27–58. 10.1146/annurev.psych.52.1.27. 11148298

[fsn370259-bib-0003] Alpro . 2024. “Die Zukunft? Sie ist Pflanzlich!” https://www.alpro.com/de/uber‐alpro.

[fsn370259-bib-0004] American Psychological Association . 2017. “Ethical Principles of Psychologists and Code of Conduct.” https://www.apa.org/ethics/code.

[fsn370259-bib-0005] American Psychological Association . 2018. “Qualitative Design Reporting Standards (JARS‐Qual).” https://apastyle.apa.org/jars/qualitative.

[fsn370259-bib-0006] American Psychological Association . 2022. “APA Dictionary of Psychology.” https://dictionary.apa.org/.

[fsn370259-bib-0007] Astolfi, M. L. , E. Marconi , C. Protano , and S. Canepari . 2020. “Comparative Elemental Analysis of Dairy Milk and Plant‐Based Milk Alternatives.” Food Control 116: 107327. 10.1016/j.foodcont.2020.107327.

[fsn370259-bib-0085] Brennan, R. L. , and D. J. Prediger . 1981. “Coefficient Kappa: Some Uses, Misuses, and Alternatives.” Educational and Psychological Measurement 41: 687–699.

[fsn370259-bib-0008] Bridges, M. 2018. “Moo‐Ove Over, Cow's Milk: The Rise of Plant‐Based Dairy Alternatives.” Practical Gastroenterology 21, no. 171: 20–27.

[fsn370259-bib-0009] Broad, G. M. , O. Zollman Thomas , C. Dillard , D. Bowman , and B. Le Roy . 2022. “Framing the Futures of Animal‐Free Dairy: Using Focus Groups to Explore Early‐Adopter Perceptions of the Precision Fermentation Process.” Frontiers in Nutrition 9: 997632. 10.3389/fnut.2022.997632. 36263302 PMC9574361

[fsn370259-bib-0010] Bundesanstalt für Landwirtschaft und Ernährung . 2024. “Milchbilanz: Erneut Weniger Milch, Käse und Butter Verbraucht.”

[fsn370259-bib-0011] Carlsson Kanyama, A. , B. Hedin , and C. Katzeff . 2021. “Differences in Environmental Impact Between Plant‐Based Alternatives to Dairy and Dairy Products: A Systematic Literature Review.” Sustainability 13, no. 22: 12599. 10.3390/su132212599.

[fsn370259-bib-0012] Coley, J. D. , and K. D. Tanner . 2012. “Common Origins of Diverse Misconceptions: Cognitive Principles and the Development of Biology Thinking.” CBE Life Sciences Education 11, no. 3: 209–215. 10.1187/cbe.12-06-0074. 22949417 PMC3433289

[fsn370259-bib-0013] Desbouys, L. , M. Rouche , K. De Ridder , C. Pedroni , and K. Castetbon . 2021. “Ten‐Year Changes in Diet Quality Among Adolescents and Young Adults (Food Consumption Survey 2004 and 2014, Belgium).” European Journal of Nutrition 60, no. 6: 3225–3235. 10.1007/s00394-021-02499-y. 33570658

[fsn370259-bib-0014] Deutsche Forschungsgemeinschaft e.V. (DFG) . 2022. “Guidelines for Safeguarding Good Research Practice. Code of Conduct.” Bonn. www.dfg.de/en.

[fsn370259-bib-0015] Deutsche Gesellschaft für Ernährung . 2021. “Einsatz von Milch und Milchprodukten in den DGE‐Qualitätsstandards im Kontext von Altersgruppen und Einer Nachhaltigen Ernährung – Wissenschaftliche Hintergründe. Bonn.”

[fsn370259-bib-0016] Deutsche Gesellschaft für Ernährung . 2024. “DGE‐Ernährungskreis.” www.dge‐ernaehrungskreis.de.

[fsn370259-bib-0017] Diethelm, K. , N. Jankovic , L. A. Moreno , et al. 2012. “Food Intake of European Adolescents in the Light of Different Food‐Based Dietary Guidelines: Results of the HELENA (Healthy Lifestyle in Europe by Nutrition in Adolescence) Study.” Public Health Nutrition 15, no. 3: 386–398. 10.1017/S1368980011001935. 21936969

[fsn370259-bib-0018] Döring, N. , and J. Bortz . 2016. Forschungsmethoden und Evaluation in den Sozial‐ und Humanwissenschaften. Springer Berlin Heidelberg.

[fsn370259-bib-0019] Dresing, T. , and T. Pehl . 2018. *Interview, Transkription & Analyse Anleitungen und Regelsysteme für Qualitativ Forschende* (8th ed.).

[fsn370259-bib-0020] Duit, R. , H. Gropengießer , U. Kattmann , M. Komorek , and I. Parchmann . 2012. “The Model of Educational Reconstruction – A Framework for Improving Teaching and Learning Science.” In Science Education Research and Practice in Europe, edited by D. Jorde and J. Dillon , vol. 13, 5th ed., 13–37. SensePublishers.

[fsn370259-bib-0021] Ercili, D. , and D. Barth . 2021. Cellular Agriculture: Lab Grown Foods. American Chemical Society.

[fsn370259-bib-0022] European Parliament , and Council of the European Union . 2013. “Regulation (EU) No 1308/2013 of the European Parliament and of the Council of 17 December 2013 Establishing a Common Organisation of the Markets in Agricultural Products and Repealing Council Regulations (EEC) No 922/72, (EEC) No 234/79, (EC) No 1037/2001 and (EC) No 1234/2007. Official Journal of the European Union.”

[fsn370259-bib-0023] European Parliament , and Council of the European Union . 2015. “Regulation (EU) 2015/2283 OF THE EUROPEAN PARLIAMENT AND OF THE COUNCIL—of 25 November 2015—on Novel Foods, Amending Regulation (EU) No 1169/2011 of the European Parliament and of the Council and Repealing Regulation (EC) No 258/97 of the European Parliament and of the Council and Commission Regulation (EC) No 1852/2001.” Official Journal of the European Union 327, no. 1: 1–22.

[fsn370259-bib-0024] European Union's Horizon 2020 Research and Innovation Programme (No 862957) . 2021a. “Plant‐Based Foods in Europe: How Big is the Market? Smart Protein Plant‐Based Food Sector Report.” www.smartproteinproject.eu.

[fsn370259-bib-0025] European Union's Horizon 2020 Research and Innovation Programme (No 862957) . 2021b. “What Consumers Want: A Survey on European Consumer Attitudes Towards Plant‐Based Foods.” www.smartproteinproject.eu.

[fsn370259-bib-0026] Federal Ministry of Food and Agriculture . 2024. “Deutschland, Wie Es Isst. Der BMEL‐Ernährungsreport 2024. Berlin.”

[fsn370259-bib-0027] Flick, U. 2018. An Introduction to Qualitative Research, edited by A. Owen , 5th ed. SAGE Publications Ltd.

[fsn370259-bib-0028] Food and Drug Administration . 2023. “Labeling of Plant‐Based Milk Alternatives and Voluntary Nutrient Statements: Guidance for Industry.”

[fsn370259-bib-0029] Formo . 2022. “Cheese Like You've Always Known.” formo.bio/cheese.

[fsn370259-bib-0030] Formo . 2024. “FAQs.” formo.bio/cheese.

[fsn370259-bib-0031] Gasteratos, K. 2019. “90 Reasons to Consider Cellular Agriculture.” http://nrs.harvard.edu/urn‐3:HUL.InstRepos:38573490.

[fsn370259-bib-0032] Geburt, K. , E. H. Albrecht , M. Pointke , E. Pawelzik , M. Gerken , and I. Traulsen . 2022. “A Comparative Analysis of Plant‐Based Milk Alternatives Part 2: Environmental Impacts.” Sustainability 14, no. 14: 8424. 10.3390/su14148424.

[fsn370259-bib-0033] Giacalone, D. , M. P. Clausen , and S. R. Jaeger . 2022. “Understanding Barriers to Consumption of Plant‐Based Foods and Beverages: Insights From Sensory and Consumer Science.” Current Opinion in Food Science 48: 100919. 10.1016/j.cofs.2022.100919.

[fsn370259-bib-0034] Gropengießer, H. 2008. “Qualitative Inhaltsanalyse in der Fachdidaktischen Lehr‐Lernforschung.”

[fsn370259-bib-0035] Gropengießer, H. 2020. “Vorstellungen im Fokus. Forschung für Verstehendes Lernen und Lehren.” In Biologiedidaktische Vorstellungsforschung. Zukunftsweisende Praxis, edited by B. Reinisch , K. Helbig , and D. Krüger , 9–25. Springer VS.

[fsn370259-bib-0036] Haas, R. , A. Schnepps , A. Pichler , and O. Meixner . 2019. “Cow Milk Versus Plant‐Based Milk Substitutes: A Comparison of Product Image and Motivational Structure of Consumption.” Sustainability 11, no. 18: 5046. 10.3390/su11185046.

[fsn370259-bib-0037] Halme, M. , A. M. Pirttilä‐Backman , and T. Pham . 2023. “The Perceived Value of Oat Milk and the Food‐Choice Motives of Young, Urban People.” British Food Journal 125, no. 13: 375–389. 10.1108/BFJ-03-2022-0238.

[fsn370259-bib-0038] Hartman Group . 2023. “Fermentation Fermenting the Future: The Growing Opportunity for Products Made With Precision Fermentation.”

[fsn370259-bib-0039] Heseker, H. , R. Dankers , J. Hirsch , et al. 2019. “Schlussbericht für das Bundesministerium für Ernährung und Landwirtschaft (BMEL). Ernährungsbezogene Bildungsarbeit in Kitas und Schulen.”

[fsn370259-bib-0040] Kempen, E. , J. Kasambala , L. Christie , E. Symington , L. Jooste , and T. Van Eeden . 2017. “Expectancy‐Value Theory Contributes to Understanding Consumer Attitudes Towards Cow's Milk Alternatives and Variants.” International Journal of Consumer Studies 41, no. 3: 245–252. 10.1111/ijcs.12331.

[fsn370259-bib-0041] Köster, E. P. 2009. “Diversity in the Determinants of Food Choice: A Psychological Perspective.” Food Quality and Preference 20, no. 2: 70–82. 10.1016/j.foodqual.2007.11.002.

[fsn370259-bib-0042] Kroeber‐Riel, W. , and A. Gröppel‐Klein . 2013. Konsumentenverhalten. 10th ed. Verlag Franz Vahlen GmbH.

[fsn370259-bib-0043] Kuckartz, U. , and S. Rädiker . 2019. Analyzing Qualitative Data With MAXQDA: Text, Audio, and Video. Springer.

[fsn370259-bib-0044] Kumar, V. 2024. International Marketing Research. Springer Nature.

[fsn370259-bib-0045] Landis, J. R. , and G. G. Koch . 1977. “The Measurement of Observer Agreement for Categorical Data.” Biometrics 33: 159–174. 843571

[fsn370259-bib-0046] Lawton, G. 2021. “Brewing Milk.” New Scientist 251: 46–49.

[fsn370259-bib-0047] Lytle, L. A. , S. Seifert , J. Greenstein , and P. McGovern . 2000. “How Do Children's Eating Patterns and Food Choices Change Over Time? Results From a Cohort Study.” American Journal of Health Promotion 14, no. 4: 222–228. 10.4278/0890-1171-14.4.222. 10915532

[fsn370259-bib-0048] Mäkinen, O. E. , V. Wanhalinna , E. Zannini , and E. K. Arendt . 2016. “Foods for Special Dietary Needs: Non‐Dairy Plant‐Based Milk Substitutes and Fermented Dairy‐Type Products.” Critical Reviews in Food Science and Nutrition 56, no. 3: 339–349. 10.1080/10408398.2012.761950. 25575046

[fsn370259-bib-0049] Markovina, J. , B. J. Stewart‐Knox , A. Rankin , et al. 2015. “Food4Me Study: Validity and Reliability of Food Choice Questionnaire in 9 European Countries.” Food Quality and Preference 45: 26–32. 10.1016/j.foodqual.2015.05.002.

[fsn370259-bib-0050] Martínez‐Padilla, E. , I. Faber , I. Lykke Petersen , and E. Vargas‐Bello‐Pérez . 2023. “Perceptions Toward Plant‐Based Milk Alternatives Among Young Adult Consumers and Non‐Consumers in Denmark: An Exploratory Study.” Food 12, no. 2: 385. 10.3390/foods12020385. PMC9858389 36673476

[fsn370259-bib-0051] Mendly‐Zambo, Z. , L. J. Powell , and L. L. Newman . 2019. “Dairy 3.0: Cellular Agriculture and the Future of Milk.” Food, Culture & Society 24, no. 5: 675–693.

[fsn370259-bib-0052] Mensink, G. B. M. , C. Lage Barbosa , and A.‐K. Brettschneider . 2016. “Prevalence of Persons Following a Vegetarian Diet in Germany.” Journal of Health Monitoring 1, no. 2: 2–14. 10.17886/RKI-GBE-2016-039. PMC9838578 36654829

[fsn370259-bib-0053] Monaco Bissonnette, M. , and I. R. Contento . 2001. “Adolescents' Perspectives and Food Choice Behaviors in Terms of the Environmental Impacts of Food Production Practices—Application of a Psychosocial Model.” Journal of Nutrition Education 33, no. 2: 72–82. 12031187 10.1016/s1499-4046(06)60170-x

[fsn370259-bib-0054] Monteiro, C. A. , G. Cannon , M. Lawrence , M. L. Costa Louzada , and P. Pereira Machado . 2019. Ultra‐Processed Foods, Diet Quality, and Health Using the NOVA Classification System. Food and Agriculture Organization of the United Nations.

[fsn370259-bib-0055] Monteiro, C. A. , G. Cannon , R. B. Levy , et al. 2016. “NOVA. The Star Shines Bright. Food Classification. Public Health.” World Nutrition 7, no. 1–3: 28–38.

[fsn370259-bib-0056] Oatly, A. B. 2024. “Oatly Sustainability Update.” https://www.oatly.com/en‐us/oatly‐who/sustainability‐plan/sustainability‐report.

[fsn370259-bib-0057] Paul, A. A. , S. Kumar , V. Kumar , and R. Sharma . 2019. “Milk Analog: Plant Based Alternatives to Conventional Milk, Production, Potential and Health Concerns.” Critical Reviews in Food Science and Nutrition 60, no. 18: 3005–3023. 10.1080/10408398.2019.1674243. 31617734

[fsn370259-bib-0058] Perfect Day Inc . 2021. “Iso‐Conformant Report. Comparative Life Cycle Assessment of Perfect Day Whey Protein Production to Dairy Protein.”

[fsn370259-bib-0059] Pointke, M. , E. H. Albrecht , K. Geburt , M. Gerken , I. Traulsen , and E. Pawelzik . 2022a. “A Comparative Analysis of Plant‐Based Milk Alternatives Part 1: Composition, Sensory, and Nutritional Value.” Sustainability 14, no. 13: 7996. 10.3390/su14137996.

[fsn370259-bib-0060] Pointke, M. , M. Ohlau , A. Risius , and E. Pawelzik . 2022b. “Plant‐Based Only: Investigating Consumers' Sensory Perception, Motivation, and Knowledge of Different Plant‐Based Alternative Products on the Market.” Food 11, no. 15: 2339. 10.3390/foods11152339. PMC9368216 35954105

[fsn370259-bib-0061] Poore, J. , and T. Nemecek . 2019. “Reducing Food's Environmental Impacts Through Producers and Consumers.” Science 363: 987–992. 10.1126/science.aaw9908. 29853680

[fsn370259-bib-0062] POSpulse GmbH . 2021. “Voll im Trend: Beliebte Milchersatzprodukte und Auffällige Marken. Über Gründe für den Konsum von Milchersatzprodukten, Liebste Produkte und Auffällige Marken.”

[fsn370259-bib-0063] Quantilope . 2024. “Welche der Folgenden ‘Milch’‐Getränke Hast du in Den Letzten 3 Monaten Konsumiert?”

[fsn370259-bib-0064] Reinders, H. 2016. Qualitative Interviews mit Jugendlichen Führen. Walter de Gruyter GmbH.

[fsn370259-bib-0065] Renner, B. , G. Sproesser , S. Strohbach , and H. T. Schupp . 2012. “Why We Eat What We Eat. The Eating Motivation Survey (TEMS).” Appetite 59, no. 1: 117–128. 10.1016/j.appet.2012.04.004. 22521515

[fsn370259-bib-0066] REWE . 2024. “Sortiment: Milchalternativen.” https://shop.rewe.de/c/milchalternativen/.

[fsn370259-bib-0067] Reyes‐Jurado, F. , N. Soto‐Reyes , M. Dávila‐Rodríguez , et al. 2021. “Plant‐Based Milk Alternatives: Types, Processes, Benefits, and Characteristics.” Food Reviews International 39: 1–32. 10.1080/87559129.2021.1952421.

[fsn370259-bib-0068] Schiano, A. , W. Harwood , P. Gerard , and M. Drake . 2020. “Consumer Perception of the Sustainability of Dairy Products and Plant‐Based Dairy Alternatives.” Journal of Dairy Science 103, no. 12: 11228–11243. 10.3168/jds.2020-18406. 33069414

[fsn370259-bib-0069] Sethi, S. , S. K. Tyagi , and R. K. Anurag . 2016. “Plant‐Based Milk Alternatives an Emerging Segment of Functional Beverages: A Review.” Journal of Food Science and Technology 53: 3408–3423. 10.1007/s13197-016-2328-3. 27777447 PMC5069255

[fsn370259-bib-0070] Shaikh, N. I. , S. S. Patil , S. Halli , U. Ramakrishnan , and S. A. Cunningham . 2016. “Going Global: Indian Adolescents' Eating Patterns.” Public Health Nutrition 19, no. 15: 2799–2807. 10.1017/S1368980016001087. 27170203 PMC10271149

[fsn370259-bib-0071] Singh‐Povel, C. M. , M. P. Van Gool , A. P. Gual Rojas , M. C. E. Bragt , A. J. Kleinnijenhuis , and K. A. Hettinga . 2022. “Nutritional Content, Protein Quantity, Protein Quality and Carbon Footprint of Plant‐Based Drinks and Semi‐Skimmed Milk in The Netherlands and Europe.” Public Health Nutrition 25, no. 5: 1416–1426. 10.1017/S1368980022000453. PMC9991740 35193730

[fsn370259-bib-0072] Statista . 2021. “Milchersatzprodukte.”

[fsn370259-bib-0073] Statista . 2024. “CONSUMERS & BRANDS Consumer Insights Report Report Overview Consumer Insights Global Methodology.”

[fsn370259-bib-0074] Swaim, J. A. , M. J. Maloni , S. A. Napshin , and A. B. Henley . 2014. “Influences on Student Intention and Behavior Toward Environmental Sustainability.” Journal of Business Ethics 124, no. 3: 465–484. 10.1007/s10551-013-1883-z.

[fsn370259-bib-0086] Szczepanski, L. , L. M. Bahlmann , C. Rötker , A. Eylering , and F. Fiebelkorn . 2025. “Attitudes toward milk alternatives and motives for (non‐) consumption: an interview study with adolescents from Germany.” BMC Nutrition. 10.1186/s40795-025-01072-8 PMC12105291 40420134

[fsn370259-bib-0075] The Good Food Institute . 2024a. 2023 State of the Industry Report Fermentation: Meat, Seafood, Eggs and Dairy.

[fsn370259-bib-0076] The Good Food Institute . 2024b. “State of the Industry Report: Cultivated Meat and Seafood.”

[fsn370259-bib-0077] UNESCO . 2017. Education for Sustainable Development Goals. United Nations Educational, Scientific and Cultural Organization.

[fsn370259-bib-0078] Verbi . 2020. MAXQDA 2020. Verbi.

[fsn370259-bib-0079] Von Koerber, K. , N. Bader , and C. Leitzmann . 2017. “Conference on “Sustainable Food Consumption” Wholesome Nutrition: An Example for a Sustainable Diet.” Proceedings of the Nutrition Society 76, no. 1: 34–41. 10.1017/S0029665116000616. 27502053

[fsn370259-bib-0080] Waltz, E. 2022. “Cow‐Less Milk: The Rising Tide of Animal‐Free Dairy Attracts Big Players.” Nature Biotechnology 40, no. 11: 1531–1533. 10.1038/s41587-022-01548-z. 36347980

[fsn370259-bib-0081] World Medical Association . 2022. “WMA Declaration of Helsinki‐Ethical Principles Formedical Research Involving Human Subjects.”

[fsn370259-bib-0082] Zhu, Q. , Y. Li , Y. Geng , and Y. Qi . 2013. “Green Food Consumption Intention, Behaviors and Influencing Factors Among Chinese Consumers.” Food Quality and Preference 28, no. 1: 279–286. 10.1016/j.foodqual.2012.10.005.

[fsn370259-bib-0083] Zollman Thomas, O. , and C. Bryant . 2021. “Don't Have a Cow, Man: Consumer Acceptance of Animal‐Free Dairy Products in Five Countries.” Frontiers in Sustainable Food Systems 5: 678491. 10.3389/fsufs.2021.678491.

[fsn370259-bib-0084] Zollman Thomas, O. , and C. Dillard . 2022. “A New Way of Making Dairy: Perceptions, Naming and Implications.”

