# Supplementary material for: “I Have No Idea What's in It!”—A Qualitative Study of Adolescents' Conceptions of Milk Alternatives
Source: Food Sci Nutr. 2025 May 20;13(5):e70259. doi: 10.1002/fsn3.70259 (PMC12121519; doi:10.1002/fsn3.70259)
Supplement: Supplementary file 1 — Appendix S1. [file FSN3-13-e70259-s001.docx]

Osnabrück University

Department of Biology/Chemistry

Biology Didactics

| Semi-structured interview guide* |
| --- |
| Milk Alternatives – Conceptions of Adolescents in Germany |

An explorative interview study with adolescents from secondary schools in the city and region of Osnabrück

*Note*. *Only the parts/questions of the interview relevant to the publication are presented.

| **Card:** 1 | **Section:** 1 - Greeting and small talk | **Intention/Research Interest:** To allow adolescents to arrive in the situation | |
| --- | --- | --- | --- |
| **Preparations**   - Place: classroom or other room in the school - Position tables and chairs - Provide drinks (water, Lemonades) and 2 glasses. - Provide cookies - Get recording device ready (2x plus batteries plus iPhone) (test recording function and prepare recording) - Two notepads, two pens, an iPad with an interview guide (as A5 index cards), a questionnaire, and working material for the respondents on the table. - Have oat drink packaging ready   **Procedure**   - Welcome - Sit down - Short small talk: - "How are you?" - "Do you feel like some water or a Lemonade?"; If so, "I'll pour you some already." - "What class did you just have?" "What interesting things did you cover in class today?" - "Have you ever participated in an interview?" - Clarify schedule:   - "Do you have any subsequent deadlines that I need to consider? The interview will take about one school hour. We could finish earlier, but it won't take longer than that." | | | |
| **Card:** 2 | **Section:** 2 - Introductions and information about the interview | | **Intention/Research Interest:**  Clarification of the general conditions and the procedure of the interview |
| **Procedure:**  (Switch on recording device 1 (iPad))   1. **Introduction**  - "Before the interview starts, I'd like to give you some information about the interview process and how I'm going to use the information. I'll start with an introduction of myself."  1. **Introduction of the Interviewer**  - "My name is Lena, and I work in biology didactics at Osnabrück University. As part of my doctoral thesis, I'm working on the topic of sustainable nutrition, and that's exactly why I'd like to talk to you today."  1. **Information on the subject and the tasks of the interview**  - Topic: Milk alternatives - Themes: Associations and consumption (reasons). - Note to adolescents: tell own thoughts and ideas, no wrong answers, ask follow-up questions.   - "More specifically, today we're going to talk about the topic of "milk alternatives." In our conversation today, I'm not interested in your knowledge on the subject of "milk alternatives". Rather, I'm interested in your ideas and thoughts on selected milk alternatives and also whether you already drink/would drink milk alternatives and why."   - "When I ask you my questions in a moment, it's important that you just tell me everything that pops into your head. There are no wrong or bad answers. I just want to know what you imagine, what you think, and why you think that when you ask each question about milk alternatives." Your ideas and thoughts about specific biology class topics help us improve our class materials and biology classes. If you have a question or ever don't understand me, you can always ask follow-up questions. Do you have a question so far?"  1. **Procedere, legal, technology**  - "Unfortunately, I can't remember everything you say, so I brought a recording device. I use it to record our conversation so that I can listen to it again later. The recording will be typed up afterwards and the recording will be deleted, since I will only continue to work with the written document for my work." - "Everything you tell me today and what we talk about will remain between us. No one will know what you said in our conversation. Neither your school administration nor your biology teacher nor your classmates will know what you said. Your name will not appear anywhere in my work. Therefore, you don't have to worry about saying something wrong." - "At any time during the call, you can communicate if you need a break or want to end the call immediately. You are welcome to put your cell phone on the table. However, it would be good if you put it on silent. If someone calls you during the interview, you can answer and/or leave the room for a short phone call. I'm just going to turn on the recorder now, but I'm not going to record anything yet. Because I'll have to ask you again in a minute if I can record our conversation so I can have your answer on tape." - "After the interview, I would like to fill out a short questionnaire with you. The questionnaire contains general questions about yourself and topics that will be addressed in the interview."  1. **Notes**    - "In between, I'll be taking notes every now and then, so I've got a pen and pad ready. On the cards here, I've written my questions that I want to ask all the students. So, I don't forget any questions, the cards are here. Every now and then I put a card aside, but don't let that confuse you. 2. **Questions, consent**     - "Do you have any questions about the interview?" (Switch on recording device 2 (recorder))    - "If you have no more questions, I will start recording now. Are you okay with that?" | | | |

| **Card:** 3 | **Section:** 3.1 – Main phase 1, Block 1.1  **Topic:** Conceptions of plant-based milk alternatives | | | **Intention/Research Interest:**  What conceptions do adolescents have of plant-based milk alternatives and their production? | |
| --- | --- | --- | --- | --- | --- |
| **Guiding Question/Narrative Prompt:**  “I've already told you that today we're talking about alternatives to cow's milk. Plant-based milk alternatives is one such alternative. What do you comprehend by plant-based milk alternatives?" (1)  “What do you think are the ingredients of plant-based milk alternatives?” You can also describe the ingredients to me using an example like oat milk.” (2)  *Intervention*  “I've brought you the packaging of oat milk. Now you can look at what an oat milk contains. However, this is just an example of a plant-based milk alternative. Take your time to look at the packaging and explain to me in your own words what a plant-based milk alternatives consists of.” (3) | | | | | |
| **Alternative wording(s):**  “How would you define plant-based milk alternatives?” (1)  “What do you think plant-based milk alternatives consist of? You are welcome to answer the question with an example.” (2)  “I've brought you the packaging of an oat milk. Now you can look at the ingredients of a plant-based milk alternative. However, this milk is just an example of a plant-based milk alternative. Take your time to look at the packaging and explain to me in your own words what a plant-based milk alternative consists of.” (3) | | | | | |
| **Content aspects:**   - General definition of “plant-based milk alternatives” (1) - Individual conceptions of the ingredients of “plant-based milk alternatives” (exemplary or general) (2) - Conceptions of “oat milk” as an example of a plant-based milk alternative (2) - Interviewer takes notes for follow-up questions. - Align learning prerequisites (3): Packaging of an oat milk; reproduction of the ingredients of an oat milk in own words | | **Further questions/notes:**   - after (1): “Can you think of any other examples of plant-based milk alternatives?” - after (2): “Can you imagine what percentage of water/oats/almond/soy (ingredient mentioned) is contained in one liter of a plant-based milk alternative?” - after (2) Transition to the conceptions of oat milk for example: “Oat milk is also an example of a plant-based milk alternative. Do you have any idea what oat drinks are made of?” - If necessary, make a comparison with cow's milk after (2), for example: “You compared plant milk alternative with cow's milk earlier. Can you go into more detail about the similarities and differences between the two liquids in terms of ingredients?” - after (3) “Leave the packaging on the table. For the other questions you can always look at the packaging if you like”. | **Intervention/Other:**   - after (2): Packaging of an oat milk - if applicable according to (2): definition of “plant milk alternatives” on request | | **Possible answers:**   - to (1): alternative to cow's milk with similar properties (e.g. appearance, consistency, taste) - to (1): plant-based alternative - to (2): Mention of the most popular raw materials such as soy, almond, oat, coconut (Pritulska et al., 2016) - to (2): Mention of water as the (main) ingredient of plant-based milk alternatives - to (3): water, oats, oil, salt - Note: different answers to questions (1) and (2) between adolescents with different diets, familiarity, consumer behavior (Haas et al., 2019; Kempen et al., 2016; McCarthy et al., 2017; Racey et al., 2017) |

| **Additional information:** **Definition of “plant-based milk alternatives”** Plant-based milk alternatives are extracts of legumes, (pseudo-)cereals, nuts or oilseeds dissolved in water, which are similar to cow's milk in terms of their appearance, texture and possible uses (Mäkinen et al., 2016; Reyes-Jurado et al., 2021; Sethi et al., 2016). Depending on the manufacturer, they may contain various additives such as stabilizers, emulsifiers, calcium or vitamins (Drewnoswki 2021; Kempen et al., 2016). |
| --- |

| **Karte: 4** | **Section:** 3.2 – Main phase 1, Block 1.2  **Topic:** Conceptions of the production of plant-based milk alternatives | | | **Intention/Research Interest:**  What conceptions do adolescents have of plant-based milk alternatives and their production? | |
| --- | --- | --- | --- | --- | --- |
| **Guiding Question/Narrative Prompt:**  “Now you know the ingredients of oat milk. “How do you imagine the production of plant-based milk alternatives such as oat milk?” | | | | | |
| **Alternative wording(s):**  “Now you know the ingredients of oat milk. Can you now explain to me how plant-based milk alternatives are produced with these ingredients?” | | | | | |
| **Content aspects:**   - Individual conceptions of the production of plant-based milk alternatives from the raw material, water, oil and salt using the example of oat drink - Observe the steps in the production of a plant-based milk alternative - Interviewer takes notes on the steps described during production | | **Further questions/notes:**   - If necessary, indicate that the production process of the liquid and not the entire value chain should be explained. - “Can you describe the sequence of the individual steps in the production process in more detail?” - “Can you describe to me again exactly which ingredient is added and processed in which step” (if necessary, adapt the statement to ingredients). - “Can you try to explain when the oil is added and why?” - “The packaging says that oat milk is heated. Try to explain to me when this step is carried out and why the liquid is heated.” - “Are there any differences in the production of a cereal-based milk alternative such as oat milk or a nut-based milk alternative such as almond milk?” - “The mixture of water, oats and oil results in a milk-like liquid at the end of the production process. Can you imagine if there are any parallels with the production process for cow's milk?” - In case of difficulties, e.g. “Can you imagine what has to happen between step X and step Y?” | **Intervention/Other:**   - Oat milk packaging - If required: Notepad for sketching | | **Possible answers:**   - Anticipated production steps: step (1), step (2), step (4), step (7) - Attempts to explain what is already known (e.g. production of cow's milk and other drinks, cooking experience, etc.) - Different levels of prior knowledge/experience with plant-based drinks (all participants) and their production (Kempen et al., 2016, Pritulska et al., 2016), e.g. due to different diets (Haas et al., 2019; Lonkila & Kaljonen, 2021; McCarthy et al., 2017) |

| **Additional information: Steps in the production of plant-based milk alternatives** Plant-based raw material e.g. pulses - (2a) raw material, dry grinding, mixing of water and ground raw material/extraction; (2b) mixing of water and raw material, soaking, wet grinding - (3) filtering of solids from the water/raw material mixture - (4) product formulation with e.g. oil, emulsifiers - (5) homogenization - (6) heat treatment - (7) enrichment with nutrients if necessary - (8) packaging  (Mäkinen et al., 2016; ProVeg, 2019; Reyes-Jurado et al., 2021) |
| --- |

| **Card: 5** | **Section:** 4 – Main phase 1, Block 2  **Topic:** Associations with plant-based milk alternatives | | | **Intention/Research Interest:**  What do adolescents associate with plant-based milk alternatives? | |
| --- | --- | --- | --- | --- | --- |
| **Guiding Question/Narrative Prompt:**  “Now I would like to know what terms come to your mind when you think of plant-based milk alternatives. What five terms come to your mind spontaneously? Please write the terms on this piece of paper. Please make sure you only write down one term per line. You have one minute to do this.” (1); *Intervention*  [...] | | | | | |
| **Alternative wording(s):**  “What do you associate with plant-based milk alternatives? On this piece of paper, write down five terms that first come to mind. Please make sure that you only write down one term per line. You have one minute to do this.” (1)  [...] | | | | | |
| **Content aspects:**   - List/name five spontaneous, unreflected associations with the term “plant-based milk alternative” (1) - Requesting an explanation for all associations mentioned to record the subjective meaning (1) | | **Further questions/notes:**   - After (1), give an indication of when the time is stopped. The interviewer stops the respondent's time. - After (1), have the respondent name the terms. - After (1): Go through the terms one by one and have the respondent explain each one (do not start with the first term here). Ask questions, for example:   - “Can you try to explain to me how you came up with these terms? Some terms are easier, some are more difficult.”   - “How did you come up with ...?”   - “Why did you come up with ...?”   - “Can you explain ... to me in more detail?”   - “Why is ... the first term that came into your head?” | **Intervention/Other:**   - Put down the prepared list (material M1) and pencil to write down the terms. | | **Possible answers:**   - to (1): Name at least three terms (mostly nouns and adjectives) as spontaneous associations from different areas, e.g:   - Diet and (trend towards) alternative lifestyles (Adamczyk et al., 2022; Laassal et al., 2019; Lemke, 2011)   - Taste (Adamczyk et al., 2022; Hamilton, 2016; McCarthy et al., 2017, Pritulska et al., 2016; Schiano et al., 2022)   - Environmental protection & animal welfare (Schiano et al., 2022, Topić & Mitchell, 2019):   - Health (McCarthy et al.,2017; Siro et al., 2008, Szakály et al., 2012; Topić, & Mitchell, 2019)   - Ingredients and nutritional value |

| **Card: 7** | **Section: 6** – Main phase 2, Block 1.1  **Topic:** Conceptions of “animal-free milk” | | | **Intention/Research Interest:**  What conceptions do adolescents have of “animal-free milk” and its production? | |
| --- | --- | --- | --- | --- | --- |
| **Guiding Question/Narrative Prompt:**  “Now you already know that today's topic is alternatives to cow's milk. A few companies are currently working on the production of a new milk alternative. This milk alternative is called “animal-free milk”. What do think “animal-free milk” is?” (1)  “What do you think are the ingredients of “animal-free milk”?” (2) | | | | | |
| **Alternative wording(s):**  “Now you already know that today's topic is alternatives to cow's milk. A few companies are currently working on the production of a new milk alternative. This milk alternative is called “animal-free milk”. Please describe to me what you think “animal-free milk is.” (1)  “Do you have any idea what “animal-free milk” consist of?” (2) | | | | | |
| **Content aspects:**   - Individual definition of “animal-free milk” (1) - Align learning requirements so that “animal-free milk” becomes clear as another option alongside plant-based milk alternatives (1) - Individual conceptions of the ingredients of “animal-free milk” (2) - Interviewer takes notes for follow-up questions. | | **Further questions/notes:**   - If reference is made to plant-based milk alternatives after (1): “Yes that's right, plant-based milk alternatives are an alternative to cow's milk, but alongside the plant-based alternatives there is “animal-free milk” as another option to cow's milk. What do you think “animal-free milk” is?” - Use ideas about the components of “animal-free milk” as a transition to production. - If the respondent has no conceptions of “animal-free milk” and its ingredients, use a direct transition to the information text, e.g.: “That's no problem at all. Then I'll give you some information about what “animal-free milk” is and how it is produced”. | **Intervention / Other:**   - Intervention according to (1) if reference is made to plant-based milk alternatives. - Provide a translation of “animal-free milk” on request. | | **Possible answers:**   - for (1): A milk alternative in the production of which no animals are involved - If applicable, a reference is made to plant-based milk alternatives - to (2): no conceptions of the ingredients of “animal-free milk” |

| **Card: 8** | **Section: 6** – Main phase 2, Block 1.2  **Topic:** Conceptions of the production of “animal-free milk” | | | **Intention/Research Interest:**  What conceptions do adolescents have of “animal-free milk” and its production? | |
| --- | --- | --- | --- | --- | --- |
| **Guiding Question/Narrative Prompt:**  “You have already told me what “animal-free milk” could consist of. Now I'd like to hear your ideas on the production of “animal-free milk”. “How do you imagine the production of “animal-free milk”?” | | | | | |
| **Alternative wording(s):**  “Do you have any ideas on how animal-free milk could be produced? Please tell me all the ideas that come into your head.” | | | | | |
| **Content aspects:**   - Individual conceptions of the production of “animal-free milk” - Interviewer makes notes for follow-up questions. - Align learning requirements so that “animal-free milk” becomes clear as another option alongside plant-based milk alternatives | | **Further questions/notes:**   - If necessary, indicate that the production process of the liquid and not the entire value chain should be explained - “Can you describe the sequence of the individual steps in the production process in more detail?” - “Can you describe to me again exactly how Z is produced with the help of X and Y” (adapt the statement to the components named by the respondent). - In case of difficulties, e.g.: “Can you imagine what is needed to produce “animal-free milk”?” - If the young people do not have any naive conceptions about the production process, there is a direct transition to the following intervention (information about “animal-free milk”) - In the case of differentiated conceptions about the production process, ask for parallels to the production of cow's milk, e.g.: “You have just explained that “animal-free milk” is like cow's milk. Can you imagine whether there are parallels to the production process of cow's milk?” | **Intervention/Other:**  / | | **Possible answers:**   - No concrete conceptions about the production process, depending on prior knowledge - Varying degrees of prior knowledge and conceptions of the production of “animal-free milk”, attempts to explain what is already known (e.g. the production of cow's milk, use of microorganisms) |

| **Card: 11** | **Section:** End | **Intention / Research Interest:**  Conclusion of the interview, clarify questions.  Supplements |
| --- | --- | --- |
| **Procedere:**   - Fade out/Reconciliation questionnaire - "Now we've talked about some things. Is there anything else you'd like to add, or do you have any questions for me?" - "Did you have any difficulties understanding during the interview? Did you find certain things particularly difficult or distracting? You're allowed to be honest with me! Your feedback might help me do something better during the next interview." - End recording - Fill out the questionnaire together (Appendix A - Part 1)   "Now I have a few final questions about your attitudes and willingness to consume milk alternatives, as well as about yourself."   - Conclusion   "Perfect, that's all the information I need. Thank you so much for participating and I hope you had a little fun. I just have one last request for you. Since I will be conducting the interview with other students at your school, it is important that you do not share any information about the topics we talked about with your classmates. You can probably imagine that it will otherwise distort the results of my study if your classmates already know the exact questions before the interview or know that the topic is "milk alternatives".   - Say thank you, hand over the voucher, have confirmation signed, and say goodbye. - Complete your own reflection questions from the short questionnaire (M4). | | |

# Literature

**Adamczyk, D., Jaworska, D., Affeltowicz, D., & Maison, D. (2022).** Plant-Based Dairy Alternatives: Consumers’ Perceptions, Motivations, and Barriers – Results from a Qualitative Study in Poland, Germany, and France. *Nutrients, 14,* 2171. https://doi.org/10.3390/nu14102171

**Haas, R., Schnepps, A., Pichler, A., % Meixner, O. (2019).** Cow Milk versus Plant-Based Milk Substitutes: A Comparison of Product Image and Motivational Structure of Consumption. *Sustainability, 11,* 5046. https://doi.org/10.3390/su11185046

**Hamilton, M. (2006).** Eating Death. *Food, Culture & Society, 9* (2), 155–177.

**Kempen, E., Kasambala, J., Christie, L., Symington, E., Jooste, L-, Van Eeden, T. (2016).** Expectancy-value theory contributes to understanding consumer attitudes towards cow’s milk alternatives and variants*. International Journal of Consumer Studies, 41*, 245–252. https://doi.org/10.1111/ijcs.12331

**Kerschke-Risch, P. (2014).** Vegan diet. Motives, approach and duration – Initial results of a quantitative sociological study. *Ernährungs Umschau International, 6*, 98–103.

**Kumar, A., & Babu, S. (2014)**. Factors Influencing Consumer Buying Behavior with Special References to Dairy Products in Pondicherry State. *International Monthly Refereed Journal of Research in Management & Technology, 3*, 65–73.

**Kurajdova, K., & Taborecka-Petrovicova, J. (2015).** Literature Review on Factors Influencing Milk Purchase Behaviour. *International Review of Management and Marketing, 5* (1), 9–25.

<https://dergipark.org.tr/en/download/article-file/366697>

**Laassal, M., & Kallas, Z. (2019).** Consumers preferences for dairy-alternative beverage using home-scan data in Catalonia. *Beverages, 5.* https://doi.org/10.3390/beverages5030055

**Lautenschlager, L., & Smith, C. (2007).** Beliefs, knowledge, and values held by inner-city youth about gardening, nutrition, and cooking. Agriculture and Human Values, 24, 245–258.

**Leitzmann, C., & Keller, M., (2013).** Vegetarische Ernährung. Eugen Ulmer.

**Lemke, H. (2011).** Klimagerechtigkeit und Esskultur – oder „Lerne Tofuwürste lieben!“. In A. Ploeger, G. Hirschfelder & G. Schönberger (Hrsg.). *Die Zukunft auf dem Tisch. Analysen, Trends und Perspektiven der Ernährung von morgen* (1. Aufl., S. 167–186). VS-Verlag & Springer

**Lonkila, A., & Kaljonen, M. (2021).** Promises of meat and milk alternatives: an integrative literature review on emergent research themes. *Agriculture and Human Values, 38*, 625–639. https://doi.org/10.1007/s10460-020-10184-9

**Mäkinen, O. E., Wanhalinna, V., Zannini, E., & Arendt, E. (2016).** Foods for special dietary needs: Non-dairy plant based milk substitutes and fermented dairy type products. *Crit. Rev. Food. Scie. Nutr*..

http//dx.doi.org/10.1080/10408398.2012.761950

**Markovina, J., Stewart-Knox, B. J., Rankin, A., Gibney, M., de Almeida, M. D. V., Fischer, A., Kuznesof, S. A., Poínhos, R., Panzone, L., & Frewer, L. J. (2015)**. Food4Me study: Validity and reliability of Food Choice Questionnaire in 9 European countries. *Food Quality and Preferences*, *45*, 26–32.

<http://dx.doi.org/10.1016/j.foodqual.2015.05.002>

**McCarthy, K. S., Parker, M-, Ameerally, A., Drake, S. L., & Drake, M., A. (2017).** Drivers of choice for fluid milk versus plant-based alternatives: What are consumer perceptions of fluid milk? *Journal of Dairy Science, 100,* 6125–6138.

**Mendly-Zambo, Z., Powell, L. J., & Newmann, L. L. (2021).** Dairy 3.0: cellular agriculture and the future of milk. *Food, Culture & Society. https://doi.org/10.1080/15528014.2021.1888411*

**Mohler, P., & Wohn, K. (2005).** Persönliche Wertorientierungen im European Social Survey. (ZUMA-Arbeitsbericht, 2005/01). Zentrum für Umfragen, Methoden und Analysen ZUMA.

<https://nbn-resolving.org/urn:nbn:de:0168-ssoar-200597>

**Palacios, O.M., Badran, J., Spence, L., Drake, M.A., Reisner, M. und Moskowitz, H.R. (2010)**. Measuring Acceptance of Milk and Milk Substitutes among Younger and Older Children. *Journal of Food Science, 75* (9), 522–526.

**Pichler, A. (2009).** Der gesundheitliche Stellenwert von Milch im Spannungsfeld zwischen wissenschaftlichem Diskurs und öffentlicher Meinung - eine qualitative und quantitative Imageanalyse. Diplomarbeit an der Universität für Bodenkultur in Wien.

**Pieniak, Z., Perez-Cueto, F., & Verbeke, W. (2013).** Nutritional status, self-identification as a traditional food consumer and motives for food choice in six European countries. *British Food Journal, 115* (9), 1297–1312. https://doi.org/10.1108/BFJ-08-2011-0198.

**Pritulska, N., Motuzka, I., Koshelnyk, A., Motuzka, O., Yashchenko. L., Jarossová, M., Krnáčová, P., Wyka, J., Malczyk, E., & Habánová, M. (2021).** Consumer Preferences on the market of plant-based milk analogues. Potravinarstvo Slovak Journal of Food Sciences, 15, 131-142. https://doi.org/10.5219/1485

**ProVeg (2019).** Pflanzenmilchreport.

https://proveg.com/de/wp-content/uploads/sites/5/2019/10/PV_Pflanzenmilch-Report_281019-final.pdf

**Racey, M., Bransfield, J., Capello, K., Field, D., Kulak, V., Machmueller, D., Preyde, M., & Newton, G. (2016)**. Barriers and Facilitators to Intake of Dairy Products in Adolescent Males and Females With Different Levels of Habitual Intake. *Childhood Obesity and Nutrition, 4*. https://doi.org./10.1177/2333794X17694227

**Reyes-Jurado, F., Soto-Reyes, N., Dávila-Rodríguez, M., Lorenzo-Leal, A. C., Jiménez-Munguía, M. T., Mani-López, E., & López-Malo, A. (2021).** Plant-Based Milk Alternatives: Types-Processes, Benefits, and Characteristics. *Food Reviews International.* https://doi.org/10.1080/87559129.2021.1952421

**Schiano, A. N., Nishku, S., Racette, C. M., & Drake, M. A. (2022).** Parent’s implicit perceptions of dairy milk and plant-based milk alternatives. Journal of Dairy Science, 105. https//doi.org/10.3168/jds.2021-21626

**Shaw, D., Grehan, E., Shiu, E., Hassan, L., Thomson, J. (2005)**. An exploration of values in ethical consumer decision making. *Journal of Consumer Behavior, 4 (3)*, 185–200. <https://doi.org/10.1002/cb.3>

**Siró, I., Kápolna, E., Kápolna, B., & Lugasi, A. (2008).** Functional food. Product development, marketing and consumer acceptance – A review. *Appetite*, 51, 456–467. <https://doi.org/10.1016/j.appet.2008.05.060>

**Steptoe, A., Pollard, T. M., Wardle, J. (1995).** Development of a measure of the motives underlying the selection of food: the Food Choice Questionnaire. *Appetite, 25* (3), 267-284. <https://doi.org/10.1006/appe.1995.0061>

**Szakály, Z., Szente, V., Kövér, G., Polereczki, Z., & Szigeti, O. (2012).** The influence of lifestyle on health behavior and preferences for functional foods. *Appetite, 58*, 406–413. https://doi.org/10.1016/j.appet.2011.11.003

**Topić, M., & Mitchell, B. (2019).** Generation Z and Consumer Trends in Environmental Packaging. Project Report. The Retail Institute, Leeds. <https://eprints.leedsbeckett.ac.uk/id/eprint/6066/>

**Van den Bergh, J., & Pallini, K. (2018).** Marketing to Generation Z. *Research World, 70,* 18–23.

<https://doi.org/10.1002/rwm3.20660>

**Wikipedia (2022-06-07)**. Milchersatz. https://de.wikipedia.org/wiki/Milchersatz

**Zibenberg, A., Greenspan, I., Katz-Gerro, T., & Handy, F. (2018).** Environmental Behavior Among Russian Youth: The Role of Self-direction and Environmental Concern. *Environmental Management, 62*, 295–304. <https://doi.org/10.1007/s00267-018-1032-7>

**Zollman Thomas, O., & Dillard, C. (2022).** A New Way of Making Dairy: Perceptions, Naming and Implications. https://prismic-io.s3.amazonaws.com/formo/79909028-2cd3-4ab2-8096-1b95f39caf38_Formo_ANewWay_ConsumerReport.pdf

## Materials for the interventions

### **Material M1: List of terms relating to “plant-based milk alternatives”**

**Five terms relating to “plant-based milk alternatives”**

|  |
| --- |
|  |
|  |
|  |
|  |
