# Supplementary material for: “I Have No Idea What's in It!”—A Qualitative Study of Adolescents' Conceptions of Milk Alternatives
Source: Food Sci Nutr. 2025 May 20;13(5):e70259. doi: 10.1002/fsn3.70259 (PMC12121519; doi:10.1002/fsn3.70259)
Supplement: Supplementary file 2 — Appendix S2. [file FSN3-13-e70259-s002.docx]

**Information text:** “**Animal-free milk**”

In Germany, more and more people are reducing their milk consumption and switching to milk alternatives. In addition to plant-based milk alternatives such as oat, soy, or almond milk, “animal-free milk” is another product that could serve as a milk alternative in the future. “Animal-free milk” mainly consists of water and cow's milk proteins. The milk proteins are responsible for the taste of cow's milk. To produce “animal-free milk”, the cow's milk proteins are not made by cows, but by microorganisms such as bacteria or yeasts. For this purpose, the cow's DNA sequences, which code for the milk proteins, are integrated into the microorganisms' DNA. The microorganisms are multiplied in a suitable culture medium and produce the cow's milk proteins. These can be further processed into “animal-free milk” or “animal-free milk”-products such as yogurt, cream cheese, or ice cream. The first animal-free dairy products have been available on the US market since 2021.

**References**

Ercili, D., & Barth. D. (2021). *Cellular Agriculture: Lab Grown Foods*. American Chemical Society.

Mendly-Zambo, Z., Powell, L. J., & Newman, L. L. (2019). Dairy 3.0: cellular agriculture and the future of milk. *Food, Culture & Society*, *24*(5), 675–693. <https://doi.org/10.1080/15528014.2021.1888411>

Zollman Thomas, O., & Bryant, C. (2021). Don’t Have a Cow, Man: Consumer Acceptance of Animal-Free Dairy Products in Five Countries. *Frontiers in Sustainable Food Systems*, *5*. https://doi.org/10.3389/fsufs.2021.678491
