# Supplementary material for: “I Have No Idea What's in It!”—A Qualitative Study of Adolescents' Conceptions of Milk Alternatives
Source: Food Sci Nutr. 2025 May 20;13(5):e70259. doi: 10.1002/fsn3.70259 (PMC12121519; doi:10.1002/fsn3.70259)
Supplement: Supplementary file 3 — Appendix S3. [file FSN3-13-e70259-s004.docx]

**Transcription rules**

Supplemented and modified transcription rules (on a content-semantic level) based on Dresing & Pehl (2018), and a tabular overview of the notations used.

1. It is transcribed word for word, not phonetically or summarized.
2. A separate paragraph is used for each speaker's contribution. A free, empty line is inserted between the speakers. Short interjections are also transcribed in a separate paragraph. A time marker is placed at the end of a speaker's contribution.
3. The interviewer is identified by an “I:”, the interviewee by a “B:” and the corresponding number of the interview, e.g. “B1:”.
4. Speaker overlaps are marked with “//”. At the beginning of the interjection, a “//” follows. The text that is spoken at the same time is then within this “//” and the other person's interjection is in a separate line and is also marked with “//”.
5. Word slurs are approximated to written German (e.g. “'n paar Fragen” becomes “ein paar Fragen”, “so 'n bisschen” becomes “so ein bisschen”, “gibt's” becomes “gibt es”).
6. Dialects are translated into High German as accurately as possible if a clear translation is possible.
7. Colloquial particles such as “ne” or “gell” are transcribed.
8. Word breaks, sentence breaks not relevant to the content, and stuttering are smoothed out or omitted. Word doublings are only noted if the content is emphasized, e.g. “That is very, very important to me”.
9. Half of the sentences that only lack completion or content-relevant sentence breaks are, however, recorded and marked with the abbreviation “/”.
10. Punctuation is smoothed in favor of readability, i.e. a period rather than a comma is used in the case of a brief lowering of the voice or ambiguous emphasis. Units of meaning should be retained.
11. Signals of understanding such as “hm”, “aha”, “ja”, and “genau”, which do not interrupt the other person's flow of speech, are generally not transcribed. They are transcribed if they are given as a direct answer to a question.
12. Filler sounds such as “um”, “uh”, “öhm”, “hm” and “mh” and short pauses (1 to 3 seconds) by the interviewer are not transcribed. Filler sounds such as “ähm”, “äh”, “öhm”, “hm” and “mh” of the interviewee(s) are transcribed.
13. After the particle “hm”, a description of the emphasis is recorded in brackets. The following can be used: affirmative, negative, reflective, questioning, comfortable, e.g. “hm (affirmative) ”.
14. Pauses are marked with “(...)”. The duration is not indicated.
15. Emotional non-verbal expressions of the speaker that support or clarify the statements (such as laughing or sighing) are placed in “()”.
16. Unintelligible words are marked with “(unv.)”. Longer incomprehensible passages should be marked with the cause, e.g. “(unv., background noise)”.
17. Data mentioned (names, places, ...) that can be used to conclude the interviewees are anonymized, i.e. removed, or changed, after the complete transcription. This is indicated in “[]”.

| Notation | Meaning |
| --- | --- |
| I: | Interviewer |
| B1:/ B2:/B3: usw. | Interviewee with interview number |
| “ähm”, “äh”, “öhm”, “hm”, “mh” | Filler sounds |
| (unv.)  (unv., Ursache) | incomprehensible words  incomprehensible words with indication of the cause |
| (lacht), (hustet), (atmet laut aus) | Non-verbal behavior or subplot |
| /  /? | Interrupting of a spoken sentence  Interrupting a spoken question |
| - | Sentence added as an afterthought; supplement (not a complete sentence) |

**Reference: Transcription rules**

Dresing, T. & Pehl, T. (2018). *Praxisbuch Interview, Transkription & Analyse. Anleitungen und 938 Regelsysteme für qualitativ Forschende*. 8th edition. Marburg.

**Redaction rules**

According to Gropengießer (2008), redaction rules and tabular overview of the notations used.

1. **Selection**

Identify the information and meaningful statements that are relevant to the question.

1. **Elimination**

Removal of filler words and redundancies by the interviewer and the interviewee: Filler words of the interviewee remain if they are used as an expression of reflection/reflection. Word duplications of the interviewee are retained if they could have a special meaning in the context of the statement, for example, the emphasis of importance.

1. **Transforming**

The dialog form of the interview is dissolved so that redigates consist of independent statements by the interviewee. Questions, references, and interjections by the interviewee are removed or integrated into the interviewee's statements.

1. **Paraphrasing**

Whole sentences are formulated. During this, the statement is grammatically smoothed, whereby the language style of the interviewee is retained. This means that violations of the generally accepted linguistic correctness of German in the areas of sentence structure, case, number, and gender are corrected. Expression or formulations of the interviewee, for example semantically inappropriate words, word-finding problems, and already established sentence constructions of the oral language are adopted.

| Notation | Meaning |
| --- | --- |
| […] | Omission of words, parts of sentences and paragraphs |
| [inserted word] | inserted words or text passages |
| for example time [expenditure] | [effort] = inserted part of a word |
| for example [is that what he means?] | [...] = inserted words or text passages that can only be assumed or inferred from the context |
| *[Additional information for the reader]* | [...] = information on interview phases, |

**Reference: Redaction rules**

Gropengießer, H. (2008). *Qualitative Inhaltsanalyse in der fachdidaktischen Lehr-Lernforschung. Die Praxis Der Qualitativen Inhaltsanalyse*.

**Notes on the rules and notations**

- The edited version is provided with general data on the interviewee using memos on the respective respondents. This includes pseudonyms, gender, age, diet, overall evaluation of animal-free milk, and willingness to consume animal-free milk.
- The redaction is provided with paragraph numbers.
- Summarized sections are marked with the beginning and end line number of the respective transcript by placing them in brackets in front of it.
- Each section is preceded by the time marker from the corresponding section of the original transcript.
- Non-verbal behavior, which is indicated in the transcript in round brackets “(...)”, is usually removed. Unless the statements must be interpreted differently considering this. Important secondary actions are presented in the form of additional information.
- Additional information on interview phases, background actions, impulses, tasks, and other relevant events are noted in italics in square brackets “[...]”.
- Omissions
  - Depending on the context, individual words, parts of sentences, whole sentences, or sections are omitted if they are irrelevant to the meaning of the statements or questions in the paper. They are marked with “[...]”. These include the verbal transition phases to the corresponding interview blocks on plant drinks and animal-free milk.
  - Statements made by the interviewee are removed without an ellipsis.
  - Filler words such as “uh, um, hm, öhm, mh” are removed without ellipsis.
  - The following filler words and redundancies are also removed without an ellipsis if they appear irrelevant to the meaning of the interviewee's statement: “halt, ja, also, dann, eigentlich, quasi, auf jeden Fall, irgendwie, irgendwo, irgendetwas, so, eben, dass” as well as "und" at the beginning of the statement and “ja, ne, nicht, sozusagen” at the end of the statement.
  - Word duplications - if redundant - are also removed without ellipsis. Unless they serve to emphasize particular facts.
  - In the case of self-corrections or clarifications, previous formulations are removed with an ellipsis “[...]”. Only final formulations are adopted.
  - Correction or replacement of individual words or parts of sentences with others: The new word or text passage is inserted first. This is followed by an ellipsis to mark the omitted words and phrases.
  - Omissions in words and/or parts of sentences are only marked if it is not a grammatical adjustment. If the omission results in a different word, this is marked with “[...]”, for example “Zeitaufwand” → Zeit [...].
- Insertions
  - If words or text passages are inserted to complete the sentences, these are noted in “[...]”.
  - Inserted words or text passages that do not emerge directly from the interviewee's statements and can only be assumed or inferred contextually are marked in “[...?]”
  - Changes or additions to individual words are only marked if they are not a grammatical adaptation of the word or sentence structure. Changes or additions that contribute to the creation of another word are marked with “[...]”.
